# Upcycling of Poly(lactic acid) Waste: A Valuable Strategy to Obtain Ionic Liquids

**DOI:** 10.1021/acssuschemeng.3c07024

**Published:** 2023-12-06

**Authors:** Giovanna Raia, Salvatore Marullo, Giuseppe Lazzara, Giuseppe Cavallaro, Sefora Marino, Patrizia Cancemi, Francesca D’Anna

**Affiliations:** † Dipartimento STEBICEF, Sezione di Chimica, Università degli Studi di Palermo, Viale delle Scienze Ed. 17 “S. Cannizzaro”, Palermo 90128, Italy; ‡ Dipartimento di Fisica e Chimica, Università degli Studi di Palermo, Viale delle Scienze Ed. 17 “S. Cannizzaro”, Palermo 90128, Italy; § Dipartimento STEBICEF, Sezione di Biologia Cellulare, Università degli Studi di Palermo, Viale delle Scienze Ed. 16, Palermo 90128, Italy

**Keywords:** poly(lactic acid), upcycling, aminolysis, lactamides, ionic liquids

## Abstract

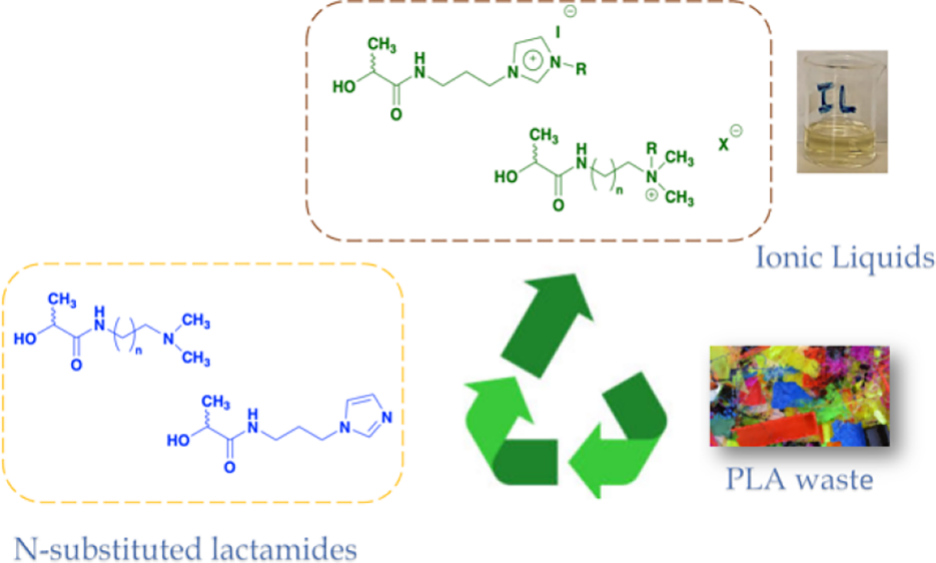

With the aim to investigate new strategies for upcycling of plastic waste, we performed aminolysis of poly(lactic acid) (PLA), using *N,N*-dimethylethylenediamine (DMEDA), *N*,*N*-dimethylpropylenediamine (DMPDA), and 3-aminopropylimidazole (API) as nucleophiles. The *N*-substituted lactamides obtained were alkylated by using alkyl halides differing in alkyl chain length, obtaining organic salts that in most cases behaved as ionic liquids (ILs). Both aminolysis of PLA and alkylation of amides were carried out taking into consideration the basic principles of the holistic approach to green chemistry, applied at a laboratory scale, and carefully selecting the nature of the reaction solvent, temperature range, and amount of reagents. Organic salts obtained from the alkylation of *N*-substituted lactamides were investigated to determine their glass or solid–liquid transitions and their thermal stability. Furthermore, cytotoxicity toward normal lung fibroblasts was also assessed. Data collected show that the proposed strategy represents a valuable protocol to upcycle plastic waste, using it as starting material to obtain alternative solvents of potential industrial relevance.

## Abstract 2

In this work, we recycle PLA by depolymerization and then we use the resulting lactamides to obtain safe ionic liquids.

## Introduction

Plastic polymers represent a class of materials that play a pivotal role in the everyday life of modern society. The large use and different fields of application make it unlikely that their substitution or banning would be necessary without lowering the standards of living. Unfortunately, the main problem directly related to the use of plastics is the environmental impact due to both production and disposal of these materials. Indeed, in some cases, the incorrect disposal of plastic waste induces accumulation in landfills and oceans, and considering that most of these synthetic polymers are designed for longevity and performance, their persistence poses serious environmental issues. A careful analysis of the actual plastic waste production allows foreseeing that it will outweigh marine fish by 2050. Furthermore, as above stated, due to the chemical inertia of plastic constituents, degradation of existing materials requires around 250–500 years, with the high risk that they can enter the human food chain through crops and animals.

Recycling is currently considered as a possible strategy to face the above problems. However, plastic production has increased exponentially from 1975, with the current worldwide annual amount of approximately 380 millions of tons, of which only about 20% is recycled. Frequently, this is a consequence of the complexity of postconsumer plastic waste having unknown composition and presenting contaminants of different nature.

Among alternative strategies suggested to partially overcome the above issues, production of biobased polymers and degradable plastics shows great promise for a green and sustainable future.^1^ This is especially important in the case of polyesters, considering not only their wide application but also their petroleum-based production. Indeed, the use of biobased raw materials could contribute to decrease the impact deriving from the production.

During the years, among biobased polymers, polylactic acid (PLA) has been proposed as the best replacement for petroleum-based polyesters and its production is currently equal to 1.23 million tons per year, representing 32% of global biodegradable plastics.^2,3^

PLA was produced for the first time in 1954 by Dupont,^4^ and its mechanical properties allow a wide range of applications ranging from food packaging to agricultural films.^5−7^ Unfortunately, although it is generally believed that biodegradable plastics have a lower environmental impact, PLA degradation occurs very slowly in the real environment, both in seawater and in soil, producing CO_2_.^8−10^ Like other thermoplastic polymers, PLA can be physically recycled by melting and reforming into new shapes. However, this kind of treatment induces a worsening of chemical and mechanical properties of the polymer, and this is the main reason why, also in this case, chemical recycling represents the more valuable strategy.

Among chemical recycling methods, hydrolysis, alcoholysis, and aminolysis have been mainly considered. Hydrolysis represents the best strategy for a closed loop, but this process requires harsh conditions and produces waste for the use of a large amount of acids or bases.^11,12^

As for alcoholysis, different cases have been reported in which Lewis acids^11,12^ or ionic liquids^13,14^ have been used as catalysts. Finally, as for aminolysis, after the first example investigating the transformation of PLA into alanine, by treatment of the polymer in ammonia solution,^15^ only few examples concerning the aminolysis of PLA, carried out in the presence of amines, have been reported.^16−18^ The main products of such processes are *N*-substituted lactamides, widely applied in the chemical industry. These substrates are normally obtained by dehydrative coupling reactions of lactic acid with amines, carried out under equilibrium conditions, producing a large amount of waste because of the removal of an acid or base catalyst.^19,20^ Then, the possibility to obtain these chemical intermediates, under mild conditions and avoiding production of a large amount of waste, represents a challenge of the current research in the field of sustainable chemistry.

**Scheme 1:**
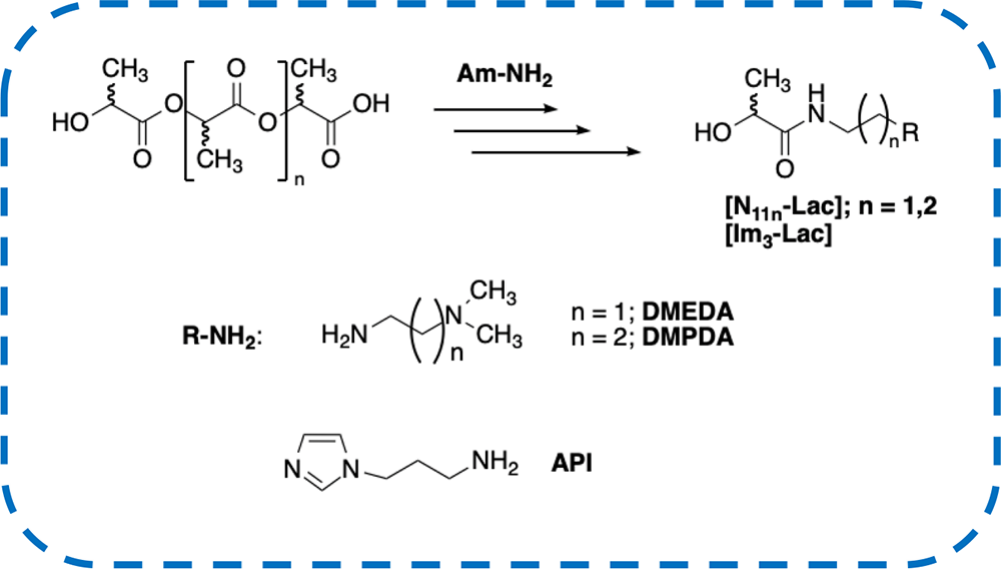
Representation of Aminolysis of PLA and Amines Used

In this paper, embracing the above challenge, we performed the aminolysis of PLA in the presence of *N,N*-dimethylethylenediamine (**DMEDA**), *N,N*-dimethylpropylenediamine (**DMPDA**), and 3-aminopropylimidazole (**API**) (Scheme 1).

Nucleophiles were chosen with the aim to evaluate the effect due to the length of the spacer between primary and tertiary nitrogens (**DMEDA** and **DMPDA**) and the one deriving from the aliphatic or aromatic nature of the tertiary amine function (**DMPDA** and **API**). In setting up the experimental conditions, we considered basic principles of the holistic approach to green chemistry,^21^ applied at a laboratory scale, with the aim to minimize energy and materials consumption.

The relevance of the above approach further increases if *N*-substituted lactamides are subsequently used as starting materials to obtain alternative solvents. To this aim, in this article, we evaluated the possibility of using the products of PLA aminolysis to prepare a new class of ionic liquids (ILs).

ILs are organic salts with a melting point lower than 100 °C, frequently liquid at room temperature and claimed as a benign and sustainable alternative to conventional organic solvents, thanks to their low vapor pressure and flammability. After pioneering papers published in the past decade of the 20th century, presenting imidazolium salts as main actors in this class of solvents, several efforts have been carried out with the aim to prepare ILs, which combine good chemical performance with low environmental cost and impact. In this context, the possibility of using raw materials as building blocks for the obtainment of ILs is a strategy widely considered in the past few years. To this aim, some examples have been reported about the preparation of ILs that originate from compounds normally existing in nature, such as amino acids, carbohydrates, carboxylic acids, and choline.^22−28^

These compounds frequently award ILs lower toxicity and higher biocompatibility. Furthermore, as they can be obtained also from biomass, they also offer the dual advantage of a lower cost, which is one of the main factors affecting ILs use on an industrial scale.

**Scheme 2:**
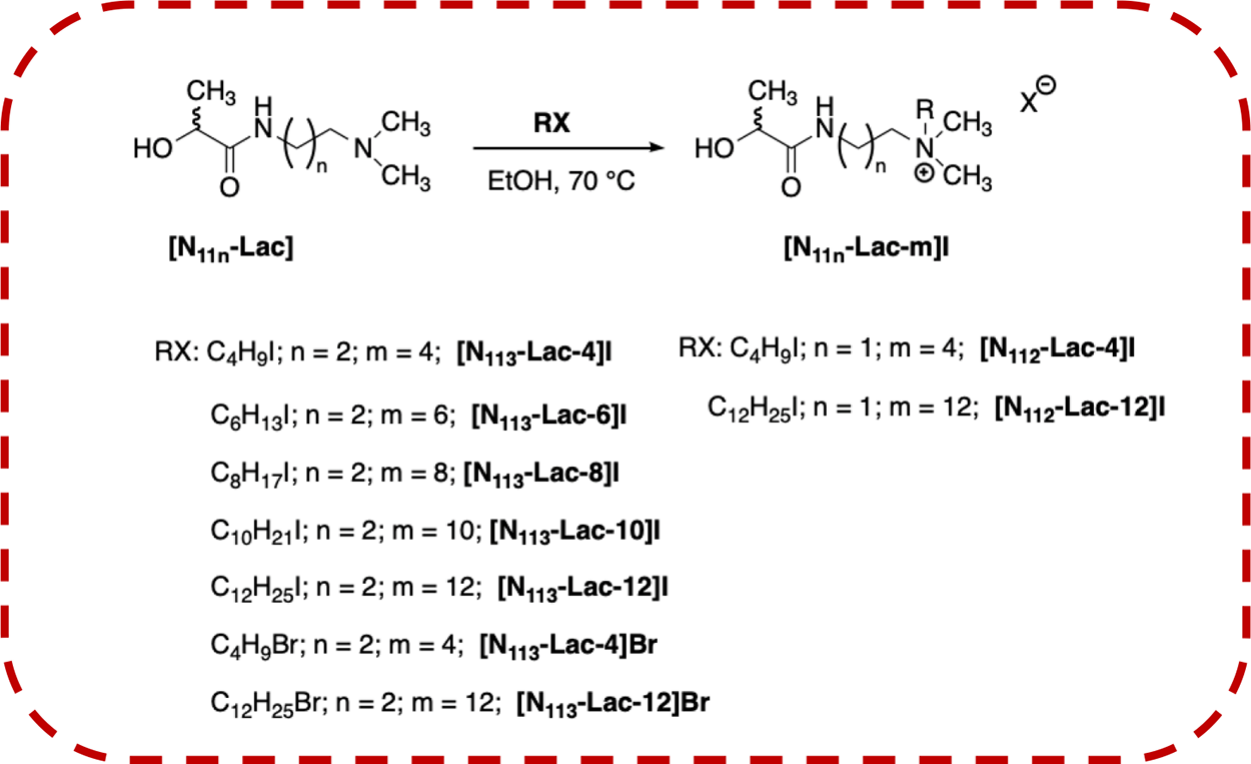
Representation of the Synthesis of Ammonium Salts

**Scheme 3:**
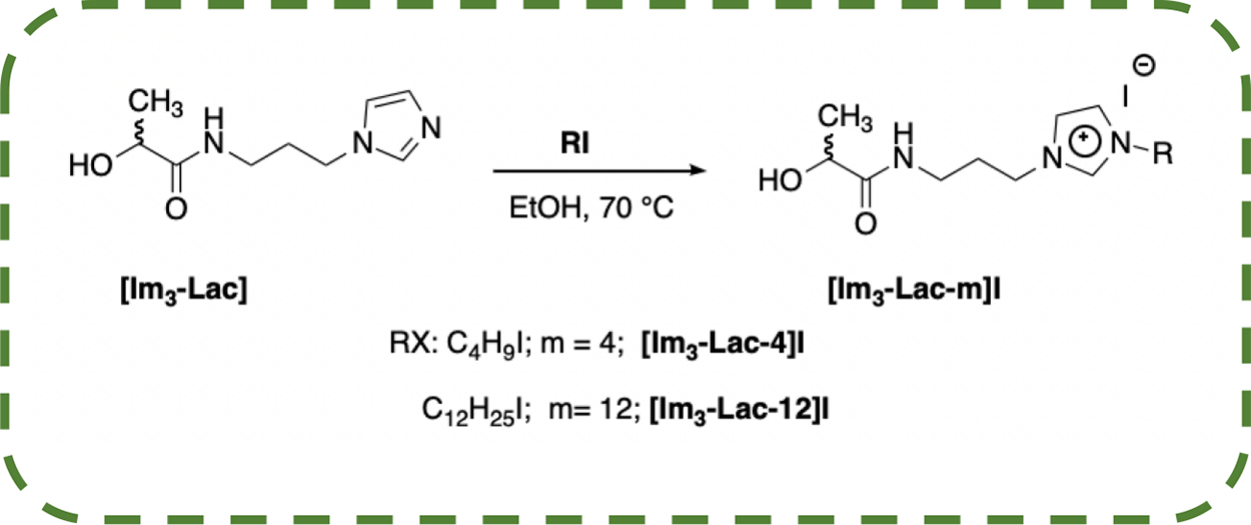
Representation of the Synthesis of Imidazolium Salts

Bearing in mind the above information and considering the structure of *N*-substituted lactamides, we evaluated the possibility of alkylating the tertiary amine function to obtain organic salts, potentially behaving as ILs (Schemes 2 and 3).

To this aim, the alkylation reaction was carried out by using alkyl iodides differing in alkyl chain length to assess the role played by the above structural feature on the properties of the salts obtained. Furthermore, the effect deriving from a different length of the spacer or from the different nature of the cationic head (ammonium or imidazolium) was also taken into consideration. We carried out synthetic procedures considering the nature of the solvent used, the amount of reagents, and also the reaction temperature, in the attempt to fully accomplish green chemistry principles.

The thermal behavior of the obtained organic salts was evaluated by performing differential scanning calorimetry (DCS) and thermal gravimetric analysis (TGA). Furthermore, considering the amide nature of the organic salts and the presence of a lactate residue on the cation structure, we also performed cytotoxicity tests by using normal lung fibroblasts.

Previous reports in the literature demonstrate that ammonium-based ILs generally show a lower toxicity with respect to the corresponding imidazolium ones. Furthermore, the toxicity of these latter can be drastically reduced if hydroxylated chains are endowed in the cation or anion structure.^28−30^ As a further point, it is also noteworthy that although different examples of ILs bearing lactate anion have been reported,^31,32^ and they have been used for extractive desulfurization of fuels,^33^ as liquid phases for CO_2_ and SO_2_ capture,^34^ and as stabilizer of metal nanoparticles,^35^ no case about the presence of lactic acid residue on the cation structure has been reported so far.

Results collected demonstrate that starting from PLA waste, it is possible to obtain lactamide-based ILs, under mild conditions and in full compliance with the holistic approach to green chemistry. Furthermore, our strategy demonstrates that a suitable combination of structural features allows us to obtain ILs with good thermal stability but very low toxicity.

## Results and Discussion

### Aminolysis of PLA

The first experiments performed were aimed at the setup of the experimental conditions. To this aim, we carried out the aminolysis of PLA, using *N,N*-dimethylpropylenediamine as nucleophile and evaluating the effect deriving from the increase in the amount of nucleophile, reaction time, and reaction temperature.

**Figure 1:**
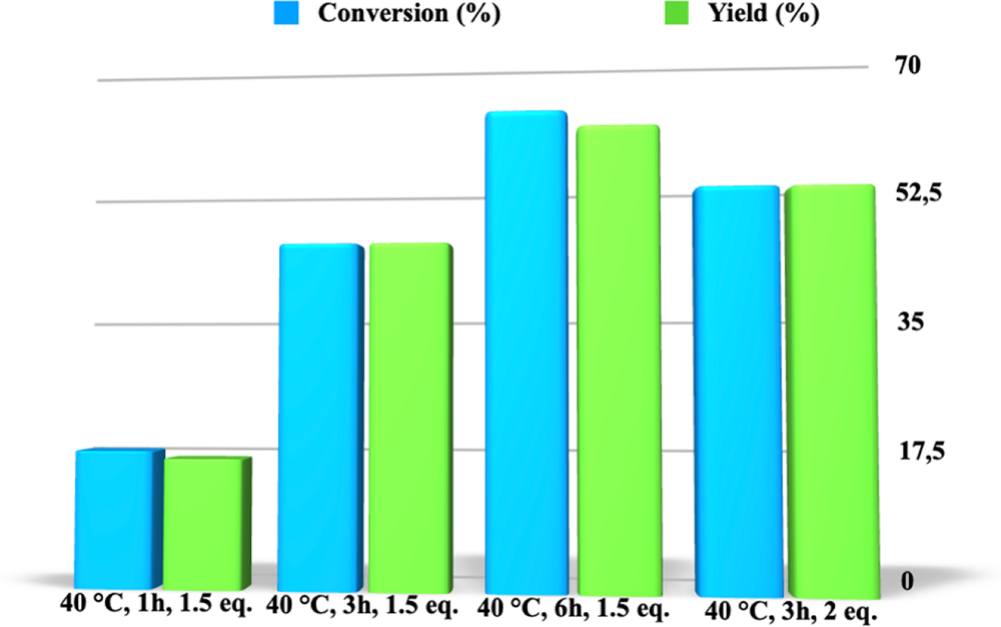
Conversion and yield values for the aminolysis of PLA carried out, at 40 °C, in the presence of *N*,*N-*dimethylpropylenediamine, as a function of time.

The first attempts were performed using 1.5 equiv of nucleophile, at 40 °C, under magnetic stirring. According to green chemistry principles, to avoid the use of unnecessary auxiliaries, we performed the reaction without using additional solvents.^36^ Then, we evaluated the effect of increasing reaction times, going from 1 up to 6 h (Figure 1 and Table S1).

In all cases, the reaction proceeded with very high selectivity, as accounted for by comparable yield and conversion values. Furthermore, we observed a gradual increase in yield from 17%, after 1 h, up to 60%, after 6 h.

With the aim to improve the performance of the process, we slightly increased the amount of nucleophile, using 2 equiv of amine at 40 °C. However, the modest increase in yield from 45 up to 52% induced us to perform the process at a lower amount of nucleophile (Figure 1).

**Figure 2:**
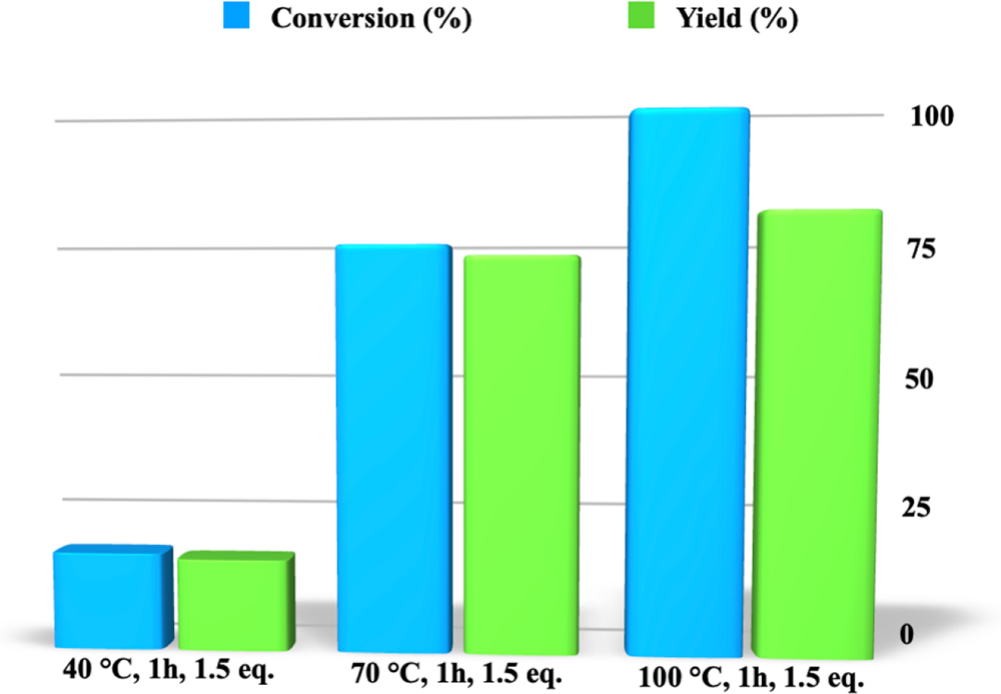
Conversion and yield values for the aminolysis of PLA carried out, for 1 h, in the presence of *N*,*N-*dimethylpropylenediamine (1.5 equiv), as a function of the temperature.

Once we determined the amount of nucleophile (1.5 equiv), we analyzed the effect deriving from the increase in temperature, performing the process in the temperature range 40–100 °C (Figure 2 and Table S1).

The gradual increase in temperature induced a parallel increase in conversion values that ranged from 18 up to 99%. However, at the highest temperature, we observed only a modest increase in yield that changed from 73 up to 81%, because of a temperature increase from 70 up to 100 °C. Conversely, a significant decrease in selectivity was detected, as accounted for by the significant difference between yield and conversion value. This is the reason that further investigation was performed at 70 °C.

**Figure 3:**
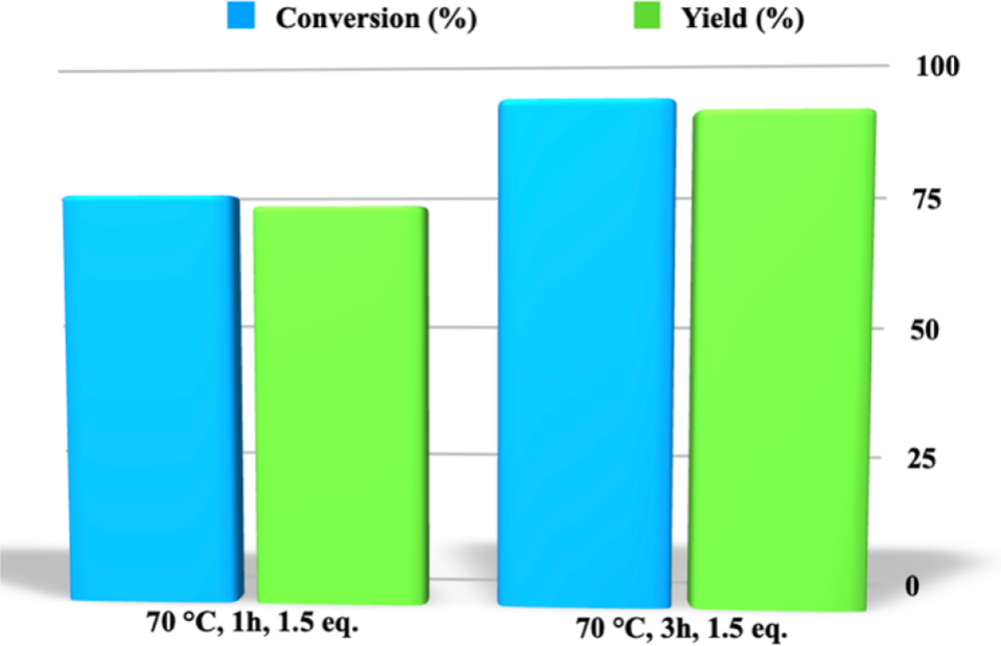
Conversion and yield values for the aminolysis of PLA carried out, at 70 °C, in the presence of *N*,*N*-dimethylpropylenediamine (1.5 equiv), as a function of time.

The last parameter we took into consideration, the temperature being the same, was the reaction time. Interestingly, going from 1 h up to 3 h, we collected very high conversion and yield values with excellent selectivity in the reaction (Figure 3 and Table S1).

**Figure 4:**
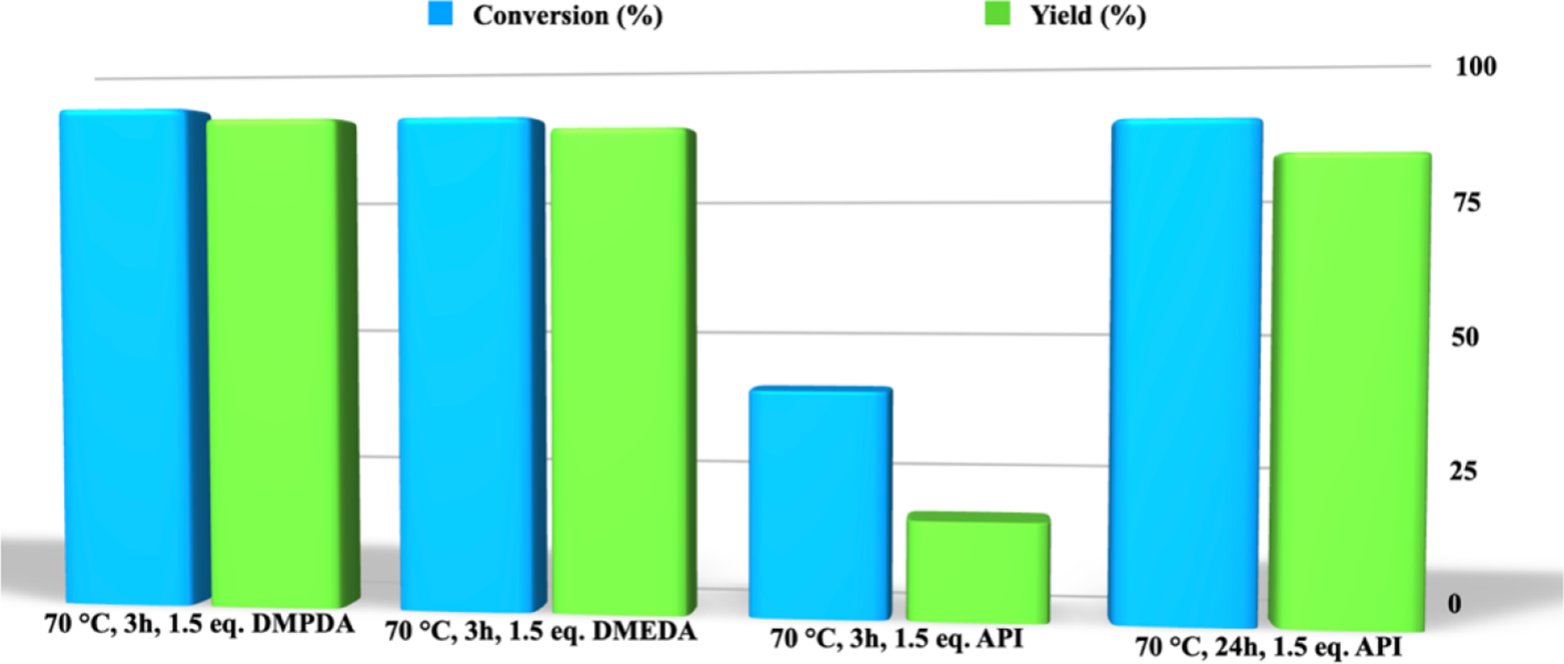
Conversion and yield values for the aminolysis of PLA carried out at 70 °C as a function of the nature of the nucleophile.

With the optimized reaction conditions in hand, we analyzed the effect derived from the nature of the nucleophile. In particular, besides *N*,*N*-dimethylpropylenediamine, we also took into consideration *N*,*N*-dimethylethylenediamine and 3-aminopropylimidazole, with the aim to explore the effect deriving from a different flexibility in the nucleophile structure, going from the propyl to ethyl spacer and, the spacer being the same, to assess the role played by the aliphatic or aromatic nature of the tertiary amine (Figure 4).

Surprisingly, we observed only a negligible effect deriving from the nucleophile flexibility, as accounted for by comparable conversion and yield values obtained by using *N*,*N*-dimethylpropylenediamine and *N*,*N*-dimethylethylenediamine. Conversely, the presence of the aromatic tertiary amine induced a significant worsening of the performance of the process. Indeed, using 3-aminopropylimidazole required extending the reaction time up to 24 h to obtain a yield higher than 89%.

As previously stated, the main aim of the present work was to perform the aminolysis of PLA in a sustainable way and, consequently, consider all the experimental parameters that could have repercussions on the environmental impact of the process. To this aim, we analyzed the results collected by using the holistic approach at green chemistry, reported some years ago by Clark et al.^21^ This kind of approach can be applied at different levels, and, as far as the laboratory scale is considered, important parameters that must be considered are, besides reaction temperature, also conversion, yield, and selectivity values. Flags of different color, green, yellow, and red, respectively, are indicative of the increase of the environmental impact of the target process. In particular, reaction temperatures in the range 0–70 °C and yield values higher than 89% are indicative of a more sustainable process.

Results obtained from the aminolysis of PLA performed under our experimental conditions as a function of the different nature of the nucleophile are reported in Table S2. Analysis of the results sheds light on the good performance of the process. Indeed, in all cases, reaction temperature perfectly complies with the guidelines of the used approach. Only in the case of 3-aminopropylimidazole, a yield value lower than 89% was collected, giving a yellow flag to the process. In all other cases, conversion, yield, and selectivity values near or higher than 90% were collected, highlighting a good compliance of the target process with the best previsions of the holistic approach. The reaction mass efficiency ranged from 63% in the case of **[Im**_**3**_**-Lac]** up to 69.6% for **[N**_**112**_**–Lac]**.

To further assess the relevance of our process, we compared our results to some of the most recently reported papers on aminolysis of PLA (Table 1).

**Table 1:** Reaction Conditions and Yields for the Aminolysis of PLA

**amine**	**catalyst**	*T* (°C)	**reaction time (min)**	**feeding ratio**	yield (%)	**reference**
DMEDA	none	70	180	1:1.5	88	this work
DMPDA	None	70	180	1:1.5	90	this work
API	none	70	24 h	1:1.5	83	this work
2-aminoethanol	none	100	60	1:4	100	(18)
benzylamine	[FeCl_2_(TMG_5_NMe_2_asme)]	60	180	1:7	99	(16)
	none	60	180	1:7	85	(16)
aniline	[N_4444_][Lac]	120	120	1:1.5	94	(17)

Our reaction conditions proved to be comparable or better than the ones recently reported in literature. Indeed, our protocol foresees the use of a lower amount of nucleophile, compared with data recently reported by Zhang et al., concerning the same process carried out in the presence of 2-aminoethanol, that gave full conversion of the polymer at 60 °C, but working with a feeding ratio PLA/amine equal to 1:4.^18^ On the other hand, our approach also exhibited better performance than the one recently reported by Liu et al. about the aminolysis of PLA carried out using aniline as nucleophile.^17^ In the above case, a yield value equal to 94% was obtained, using a comparable amount of nucleophile (1.5 equiv) but operating at 120 °C. On the other hand, our conditions proved to be significantly better than the ones applied by Herres-Pawlis et al. that achieved a quantitative yield, at 60 °C in the presence of a guanidine Iron(II) catalyst but using the nucleophile with a feeding ratio equal to 1:7.^16^

### Alkylation of N-Substituted Lactamides

After the optimization of the reaction conditions for the aminolysis of PLA, with the aim to obtain products having potential industrial value, we considered the possibility to alkylate the tertiary amine function of the amides (Schemes 2 and 3) for the obtainment of ammonium salts that, depending on their melting point, could behave as ILs.

We optimized the reaction conditions using **[N**_**113**_**–Lac]** as substrate and butyl or dodecyl iodide as alkylating agent to assess the effect of the alkyl chain length on the performance of the process. We carried out the reaction at 70 °C, which represents a temperature value that perfectly resides in the range 0–70 °C, indicated as relatively mild conditions according to the holistic approach.^21^

First attempts were carried out by using 1.5 equiv of alkyl halides in solvent-free conditions. However, in all cases, reaction mixtures appeared as biphasic systems and after 24 h (butyl iodide) or 72 h (dodecyl iodide), we did not observe product formation. Consequently, we used ethanol as solvent that, according to solvent selection guides, is classified as a recommended solvent,^37^ operating in a very concentrated solution. Indeed, 100 mg of amide was solubilized in 2 mL of EtOH and the alkyl iodide was added dropwise. Alkylation reactions were performed changing the amount of alkyl iodide from 1 up to 1.5 eq. (Table S3). However, data collected demonstrate that they proceeded with a full conversion only using 1.5 equiv of alkyl iodide.

Taking into consideration the above results, we changed the length of the alkyl chain and the spacer to evaluate the effect of the above structural parameters on the process performance. Reaction times ranged from 24 up to 72 h, in dependence of the alkyl chain and the amide nature (see Experimental Section in the SI). In general, lower reaction times were used for butyl chain and hexyl derivatives whereas the further lengthening induced a corresponding increase in the reaction time. The longest reaction time (2 weeks) was used in the case of **[Im**_**3**_**-Lac-12]I**.

In all cases, we obtained a full conversion and yields ranging from 83% (**[N**_**113**_**–Lac-10]I**) to 97% (**[N**_**113**_**–Lac-6]I** and **[Im**_**3**_**-Lac-12]I**). Results are reported in Table 2, together with reaction mass efficiency values (RMI).

**Table 2:** Yields and Reaction Mass Efficiency (RME) for the Alkylation Reaction of the *N*-Lactamides

**ammonium salt**	yield (%)^a^	RME (%)
**[N**_**112**_**-Lac-4]I**	92	72
**[N**_**113**_**-Lac-4]I**	95	75
**[N**_**113**_**-Lac-6]I**	97	76
**[N**_**113**_**-Lac-8]I**	98	76
**[N**_**113**_**-Lac-10]I**	83	63
**[N**_**113**_**-Lac-12]I**	93	70
**[N**_**112**_**-Lac-12]I**	92	69
**[Im**_**3**_**-Lac-4]I**	99	74
**[Im**_**3**_**-Lac-12]I**	97	79
		
**[N**_**113**_**-Lac-4]Br**	91	74
**[N**_**113**_**-Lac-12]Br**	70	53

a Yields were reproducible within ±2.0%.

With the only exception of **[N**_**113**_**-Lac-10]I**, the yield and selectivity were higher than 91%, giving a green flag to the process. In all cases, solvent used to perform the reaction, as well as the ones used in the workup procedures, can be easily recycled. Consequently, reaction mass efficiency shows good values ranging from 70 up to 79%.

**Figure 5:**
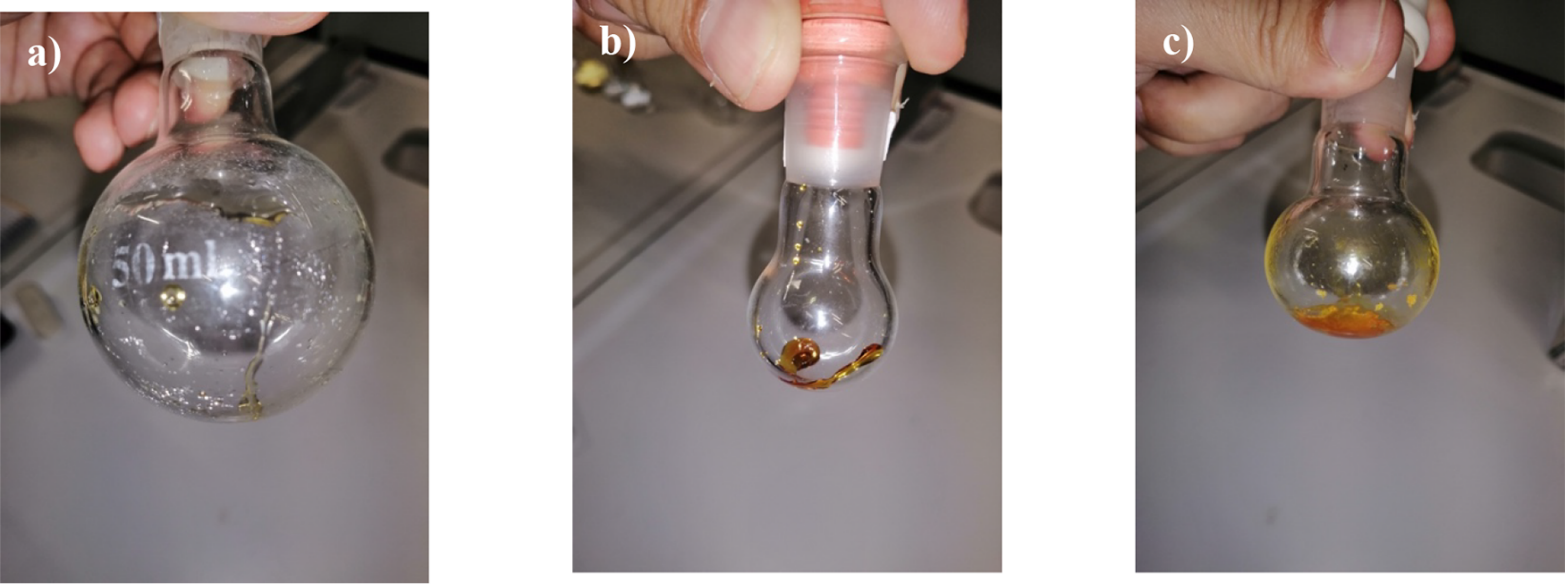
Picture of the obtained organic salts: (a) **[N**_**112**_**-Lac-4]I**; (b) **[N**_**113**_**-Lac-6]I**; (c) **[N**_**112**_**-Lac-12]I**.

With the only exceptions being **[N**_**113**_**-Lac-4]I** and **[N**_**113**_**-Lac-4]Br**, all organic salts behaved as viscous liquids at room temperature. Some representative pictures are listed in Figure 5.

To have information about the effect of the nature of the anion on the properties of the organic salts, also butyl and dodecylbromides of *N*-lactamide deriving from *N,N*-dimethylpropylenediamine were prepared (**[N**_**113**_**-Lac-4]Br** and **[N**_**113**_**-Lac-12]Br**; Scheme 2). These were obtained under the same reaction conditions, used for the alkylation, but only after reacting the amide and corresponding bromides for 96 and 72 h, respectively. At the end of the reaction time, also in this case, we recorded a full conversion but with significantly different yield values that were equal to 70 and 91% in the case of [**N**_**113**_**-Lac-12]Br** and **[N**_**113**_**-Lac-4]Br**, respectively. As a consequence of the low yield, in the case of **[N**_**113**_**-Lac-12]Br**, we obtained the lowest reaction mass efficiency that was equal to 53% (Table 1).

In addition, we investigated the possibility of recovering excess halide. To this aim, we evaporated the organic phase obtained after the washing step in the workup of the synthesis of **[N_113_-Lac-12]I**, as a representative case. The ^1^H NMR spectrum of the residue, reported in Figure S5, shows that it is practically constituted by only the alkyl iodide, suggesting the concrete possibility to recover it.

### Thermal Behavior

Organic salts were first studied using DSC measurements. Melting or glass transitions are reported in Table S4, whereas DSC traces are displayed in Figure S1. In most cases, in the used temperature range (−40–60 °C), on heating, we did not observe transitions, confirming the ionic liquid nature of the organic salts. Melting processes were detected only in the case of **[N**_**112**_**-Lac-4]I**, **[N**_**113**_**-Lac-4]I**, **[N**_**113**_**-Lac-4]Br**, and **[Im**_**3**_**-Lac-12]I**, and, among the above cases, only **[N**_**112**_**-Lac-4]I** and **[Im**_**3**_**-Lac-12]I** showed transition at temperature values lower than 100 °C. Data collected clearly evidence that transition temperature increased by increasing the spacer length (*T*_m**[N113-Lac-4]I**_ > *T*_m**[N112-Lac-4]I**_) but also changing the anion nature (*T*_m**[N113-Lac-4]Br**_ > *T*_m**[N113-Lac-4]I**_).

Thermal stability of all of the organic salts was investigated by TGA measurements. TGA traces are reported in Table S1. With the only exception of **[N**_**113**_**-Lac-4]Br** and **[N**_**113**_**-Lac-12]Br**, in all of the other cases, we observed a single-step degradation process. In all cases, we considered the temperature corresponding to the first degradation process, as the short-term stability limit, as the highest temperature ILs can withstand for short periods. These temperature values are reported in Table 3.

**Table 3:** Decomposition Temperatures Obtained for ILs

	*T*_**ons**_**(1) (°C)**	**Δ***T***G peak(1) (°C)**	*T*_**ons**_**(2) (°C)**		*T*_**ons**_**(1) (°C)**(25)
**[N**_**112**_**-Lac-4]I**	230.2	252.13			
**[N**_**112**_**-Lac-12]I**	218.23	250.21		**[N**_**112**_**-Glu-12]I**	210.3
**[N**_**113**_**-Lac-4]I**	261.01	295.76		**[N**_**113**_**-Glu-4]I**	191.7
**[N**_**113**_**-Lac-6]I**	234.34	264.57			
**[N**_**113**_**-Lac-8]I**	238.92	272			
**[N**_**113**_**-Lac-10]I**	230.15	261.13			
**[N**_**113**_**-Lac-12]I**	235.17	263.41			
**[Im**_**3**_**-Lac-4]I**	292.05	320.8		****[Im**_**3**_-Glu-4]I**	197.3
**[Im**_**3**_**-Lac-12]I**	274.24	310.33			
					
**[N**_**113**_**-Lac-4]Br**	251.2	277.9	457.1		
**[N**_**113**_**-Lac-12]Br**	226.7	245.3	466.4		

Decomposition temperatures of ILs ranged from 245.3 (**[N**_**113**_**-Lac-12]I)** up to 320.8 °C (**[Im**_**3**_**-Lac-4]I).** Thermal stability significantly decreased by lengthening the alkyl chain. On the other hand, alkyl chain and anion being the same, it increased on going from **[N**_**112**_**-Lac-4]I** to (**[N**_**113**_**-Lac-4 ]I** and from **[N**_**112**_**-Lac-12]I** to **[N**_**113**_**-Lac-12]I**, according to the increase in the alkyl spacer length, with Δ*T* depending on the length of the alkyl chain (Δ*T* = 43.7 and 13.2 in the case of butyl and dodecyl derivatives, respectively).^25^ This result was different from the one we collected for *N*-glucosamide-based ILs. Indeed, in that case, we observed a decrease in thermal stability on going from an ethyl to propyl spacer.

Comparison between data collected for bromide (**[N**_**113**_**-Lac-4]Br** and **[N**_**113**_**-Lac-12]Br**) and iodide salts (**[N**_**113**_**-Lac-4]Br** and **[N**_**113**_**-Lac-12]I**) evidenced a higher thermal stability of the iodides than bromide salts. This result perfectly accounts for the increase in thermal stability induced by a decrease in the anion nucleophilicity previously reported for imidazolium salts.^38^

Finally, data were collected accounting for the higher thermal stability of imidazolium with respect to the corresponding ammonium salts (*cfr.***[N**_**113**_**-Lac-4]I/[N**_**113**_**-Lac-12]I** and [**Im**_**3**_**-Lac-4]I/**[**Im**_**3**_**-Lac-**_**12**_**]I**), in accordance with a previous observation in literature comparing imidazolium and ammonium salt having alkyl chains of the same length.^39^

In order to analyze the effect of the oxygenated chain on the thermal stability, we compared decomposition temperatures, at 95% wt, for *N*-glucosamides-based ILs^25^ with the one now obtained for *N*-lactamide-based ILs (Table 2). In general, the above comparison evidenced a higher thermal stability for *N*-lactamides-based ILs with respect to the corresponding *N*-glucosamide-based ones.

### Biological Activity of ILs: Morphological Assessment

**Figure 6:**
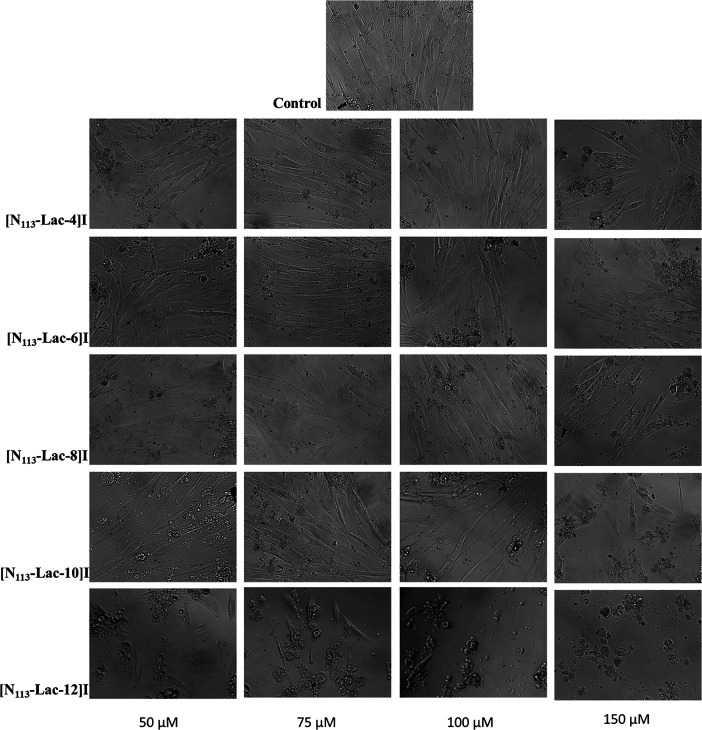
Micrographs performed by inverted phase-contrast microscopy of IMR-90 cells treated for 24 h with different concentrations (50, 75, 100, 150 μM) of **[N**_**113**_**-Lac-4]I, [N**_**113**_**-Lac-6]I**, **[N**_**113**_**-Lac-8]I**, **[N**_**113**_**-Lac-10]I**, and **[N**_**113**_**-Lac-12]I**. Magnification 200×.

**Figure 7:**
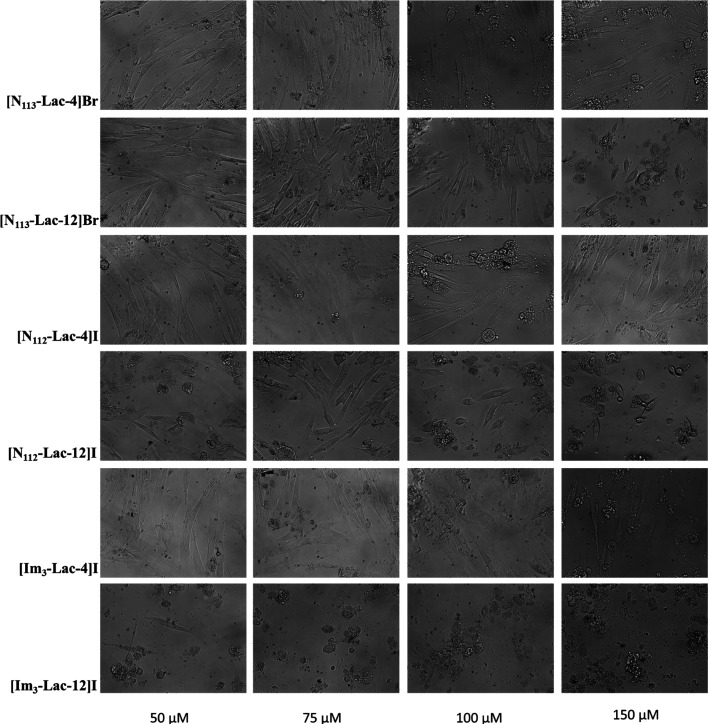
Micrographs performed by inverted phase-contrast microscopy of IMR-90 cells treated for 24 h with different concentrations (50, 75, 100, 150 μM) of **[N**_**113**_**-Lac-4]Br**, **[N**_**113**_**-Lac-12]Br**, **[N**_**112**_**–Lac-4]I**, **[N**_**112**_**-Lac-12]I, [Im**_**3**_**-Lac-4]I**, and **[Im**_**3**_**-Lac-12]I**. Magnification 200×.

A morphological assessment of human fetal lung fibroblasts (IMR-90 cells) by a phase-contrast inverted microscope was employed, monitoring the cells under an inverted light microscope after 24 h of treatment with different concentrations (50, 75, 100, 150 μM) of ILs (Figures 6 and 7). IMR-90 control cells maintained their original morphology consisting of elongated and slender forms and the strikingly regular form, with longitudinal alignment of cells. At a lower concentration of treatments (50 μM), the ILs bearing short alkyl chains showed weaker biological effectiveness compared to those with long apolar alkyl chains in terms of morphological changes and cytotoxic effects. This lower biological activity was maintained at higher concentrations of treatments, especially for salts bearing butyl or hexyl chains. The obtained results clearly indicate that, according to the specific fields of application, the biological activity of ILs is quite tunable, by modifying structural characteristics.

### Structure vs Cytotoxicity

**Figure 8:**
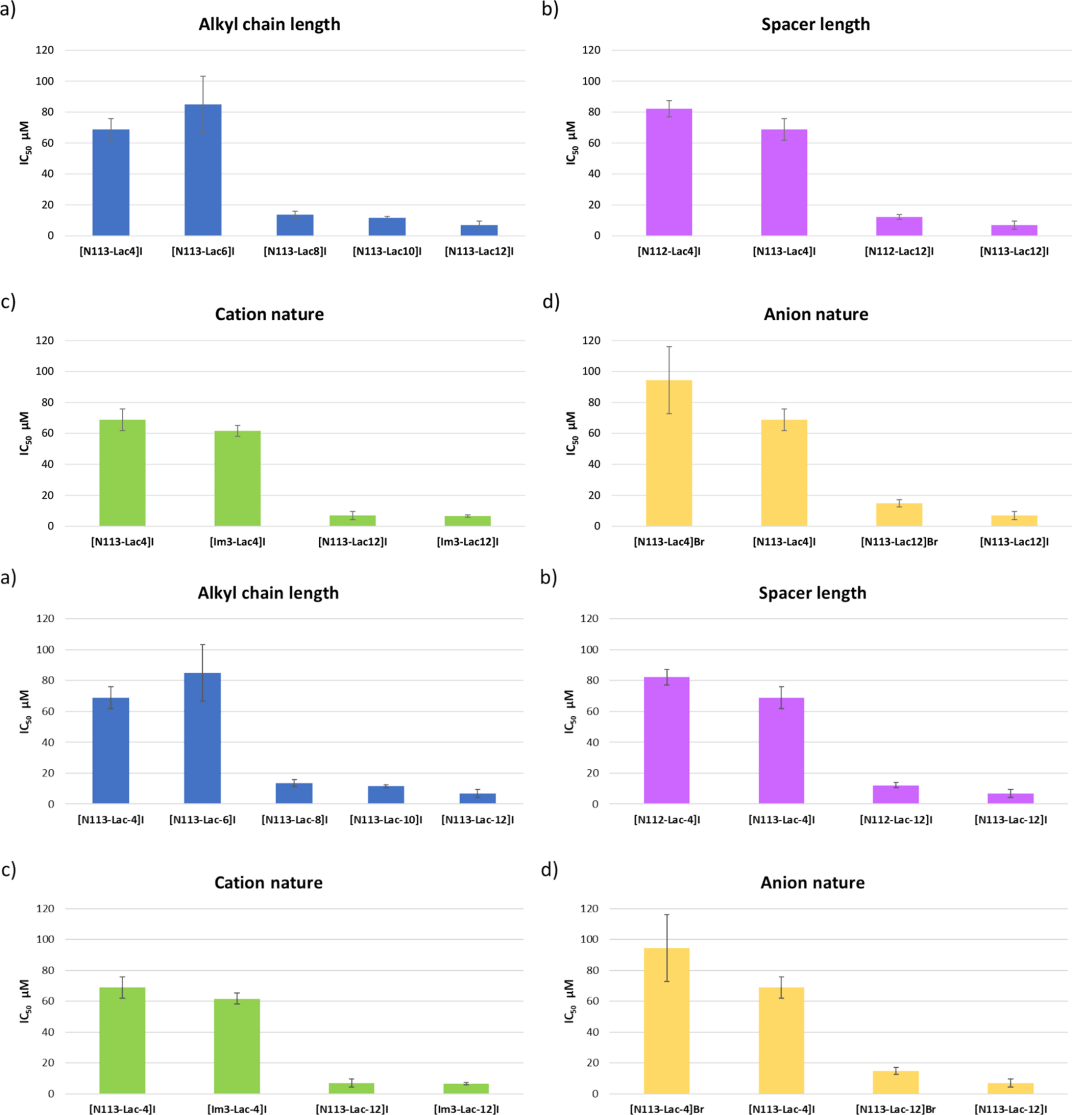
Histograms showing IC_50_ values expressed as μM concentration of ILs sorted for different structural characteristics: (a) alkyl chain length in the cation (**[N**_**113**_**-Lac-4]I**, **[N**_**113**_**-Lac-6]I**, **[N**_**113**_**-Lac-8]I**, **[N**_**113**_**-Lac-10]I**, and **[N**_**113**_**-Lac-12]I**), (b) spacer length (**[N**_**112**_**-Lac-4]I**, **[N**_**113**_**-Lac-4]I**, **[N**_**112**_**-Lac-12]I**, and **[N**_**113**_**-Lac-12]I**), (c) cation nature (**[N**_**113**_**-Lac-4]I**, **[Im**_**3**_**-Lac-4]I**, **[N**_**113**_**-Lac-12]I**, **[Im**_**3**_**-Lac-12]I**), and (d) anion nature (**[N**_**113**_**-Lac-4]Br**, **[N**_**113**_**-Lac-4]I**, **[N**_**113**_**-Lac-12]Br**, and **[N**_**113**_**-Lac-12]I**).

The cytotoxicity of ILs was evaluated by using a 24 h toxicity assay. The IC_50_ values were calculated using a dose–response model by means of sigmoidal fitting of curves of percent inhibition versus logarithm of tested concentrations. In all cases, the MTT assay was performed using diluted 1 × 10^–3^ M stock solutions of salts. The results obtained are gathered in Table S3 and depicted in Figure 8.

IC_50_ values range from 6.58 μM up to 94.4 μM. In general, these organic salts prove to be less toxic than *N*-gluconamide-based ILs, which exhibited IC_50_ values ranging from 0.06 μM up to 12.6 μm.^25^

Data collected will be analyzed as a function of different structural features of the salts.

First, as can be seen from Figure 8a, butyl and hexyl derivatives represent the terms of lower toxicity. The further increase in alkyl chain length induces a sharp increase in cytotoxicity.^40^ These results are consistent with data previously reported in literature and can be easily ascribed to the increase in the lipophilicity that induces a better interaction with the phospholipid bilayers of biological membranes.^41^

On the alkyl chain being the same, small changes were detected as a function of the length of the alkyl spacer. In particular, ILs bearing the ethyl spacer are slightly less toxic than the ones featured by the presence of the propyl spacer (Figure 8b). This result is different from the one we collected studying the biological activity of *N*-glucosamide-based ILs.^25^

Interestingly, the presence of the *N*-lactamide unit induced comparable toxicity in imidazolium- and ammonium-based salts, as accounted for by the comparison between IC_50_ values detected for **[Im**_**3**_**-Lac-4]I** and **[N**_**113**_**-Lac-4]I**, as well as the ones for **[Im**_**3**_**-Lac-12]I** and **[N**_**113**_**-Lac-4]I** (Figure 8c). This results appear quite surprising considering the frequently claimed lower toxicity of aliphatic ILs with respect to the corresponding aromatic ones. This finding clearly supports the one previously reported in literature about the role played by oxygenated chains in decreasing the IL toxicity.^42^

Finally, independently from the cation structure, bromide salt exhibited lower toxicity with respect to the corresponding iodide salts (Figure 8d). Probably, the above result can be related to the anion lipophilicity, as accounted for by its ability to interact with water molecules. Bromide, as a consequence of its higher charge density, should better interact with water molecules exhibiting a lower lipophilicity and consequently a lower ability to interact with the lipophilic membrane. This hypothesis is also supported by data previously reported in literature about the ability of the anion to permeate phospholipidic bilayers and showing a higher permeation rate for the iodide anion with respect to the bromide one.^43^

## Conclusions

With the aim to upcycle plastic waste and to realize an open loop that starting from waste materials allows the obtainment of products of industrial values, like alternative solvents, we performed aminolysis of PLA, under sustainable conditions. In particular, temperature and time of reaction, as well as the amount and nature of nucleophile, were carefully evaluated, to minimize the environmental impact of the process. This allowed the obtainment of *N*-substituted lactamides, avoiding harsh conditions and the use of a large amount of acidic or basic catalysts.

A careful analysis of the *N*-lactamide structure sheds light on the possibility of using them as starting materials for the preparation of organic salts, potentially behaving as ionic liquids. To this aim, they were successfully alkylated, using alkyl halides differing for the alkyl chain length and the halide nature. Proposed synthetic protocols perfectly fit the guidelines of the holistic approach to green chemistry and allowed obtaining a green or yellow flag for related parameters, like conversion and yield, while RMI values ranged from 52 up to 79%.

The majority of the ammonium salts obtained were viscous liquids at room temperature, behaving as ILs. Properties of these solvents, like melting temperature, thermal stability, and toxicity toward normal lung fibroblasts, were studied as a function of different structural features, showing good thermal stability and toxicity lower than the one recently detected for some *N*-glucosamide-based ILs.^25^ Interestingly, the results collected demonstrate how careful modulation of structural features, like alkyl chain and spacer length, as well as anion and cationic head nature allows the obtainment of ILs of good thermal stability and low toxicity. To the best of our knowledge, this is the first report about the obtainment of ILs from plastic waste, featured by a lactate residue on the cation structure. The presence of the above residue should award these ILs a good coordination ability, and also thanks to the good thermal stability and low toxicity, they could be tested, in the future, for extractive desulfurization of fuels or as liquid phases for CO_2_ and SO_2_ capture. Furthermore, according to previous reports, iodide-based ammonium salts are frequently indicated as good corrosion inhibitors.^44−46^ Consequently, the proposed strategy in this work could represent a suitable way to upcycle waste for the obtainment of useful industrial intermediates.

## Supporting Information Available

Experimental procedures, yields and conversions for PLA aminolysis, IC_50_ values, thermogravimetric curves, ^1^H and ^13^C NMR spectra, and FTIR spectra of lacatamide-based ionic liquids (PDF)
