# Supplementary material for: Upcycling of Poly(lactic acid) Waste: A Valuable Strategy to Obtain Ionic Liquids
Source: ACS Sustain Chem Eng. 2023 Dec 6;11(50):17870–80. doi: 10.1021/acssuschemeng.3c07024 (PMC10732281; doi:10.1021/acssuschemeng.3c07024)
Supplement: Supplementary file 1 — sc3c07024_si_001.pdf [file sc3c07024_si_001.pdf]

Supporting Information for
Upcycling of Poly(lactic acid) waste: A valuable strategy to obtain Ionic Liquids

*Giovanna Raia, Salvatore Marullo, Giuseppe Lazzara, Giuseppe Cavallaro, Sefora Marino, Patrizia Cancemi, Francesca D'Anna**

[a]Università degli Studi di Palermo, Dipartimento STEBICEF, Sezione di Chimica, Viale delle Scienze Ed. 17 "S. Cannizzaro", 90128 Palermo, Italy.
[b]Università degli Studi di Palermo, Dipartimento di Fisica e Chimica, Viale delle Scienze Ed. 17 "S. Cannizzaro", 90128 Palermo, Italy.
[c]Università degli Studi di Palermo, Dipartimento STEBICEF, Sezione di Biologia Cellulare, Viale delle Scienze Ed. 16, 90128 Palermo, Italy.

Email: francesca.danna@unipa.it

Number of Pages: 29

Number of Tables: 5

Number of Figures: 5

Experimental Section **Pag. S1-S6**

Table S1. Optimisation of reaction condition for the aminolysis of PLA in the presence of *N,N*-dimethylpropylenediamine. **Pag. S6**

Table S2. Conversion, yield and selectivity values for the aminolysis of PLA in the presence of different nucleophiles. Results are evaluated on the ground of the holistic approach to Green Chemistry (Flag).[2] **Pag. S6**

Table S3. Optimisation of reaction condition for the alkylation of **[N_{113}-Lac]** in the presence of butyl or dodecyl iodide at 70 °C, for 24h. **Pag. S7**

Table S4. Glass transition (T_g) and melting temperature (T_m), thermodynamic parameters determined by DSC measurements relevant to salt synthesised. **Pag. S7**

Table S5. IC_{50} values relevant to the ILs obtained. **Pag. S8**

Figure S1. DSC traces of lactamide-based ionic liquids. **Pag. S9-S10**

Figure S2. Thermogravimetric curves of lactamide-based ionic liquids. **Pag. S11**

Figure S3. ^1H and ^{13}C NMR spectra of lactamide-based salts. **Pag. S12-S24**

Figure S4. FTIR spectra of lactamide-based salts. **Pag. S25-S26**

Figure S5. ^1H spectrum in $CDCl_3$ of the residue of alkylation, to recover iodododecane. **Pag. S27**

Experimental Section

Materials

Polylactic acid (PLA), *N-N*-dimethyl-1,3-propanediamine, *N-N*-dimethyl-1,2-ethylenediamine, 1-(3-aminopropyl) imidazole, 1-iodobutane, 1-iodohexane, 1-iodooctane, 1-iododecane, 1-iodododecane, 1-bromobutane, 1-bromododecane, methanol, ethanol and ethyl acetate were obtained from commercial sources and used without further purification.

General procedure for the aminolysis of PLA

Polylactic acid (PLA) and the suitable amine (1.5 eq), were put in a round bottom flask the mixture obtained was and heated to 70 °C. The reaction time for each aminolysis is reported below.
Subsequently, unreacted PLA was filtered off and washed with 15 mL of ethyl acetate. Unreacted PLA was dried and weighed. The filtrate was under reduced pressure:

$$\text{Conversion}_{\text{PLA}} = \frac{(\text{PLA initial weight - PLA residual weight})}{\text{PLA initial weight}} \cdot 100 \qquad (1)$$

$$\text{Yield} = \frac{\text{Amide initial weight}}{\text{Amide theoretical weight}} \cdot 100 \qquad (2)$$

**Lactamide**	**Reaction time (h)**
N-[2-(dimethylamino)ethyl]-2-hydroxypropanamide	3
N-[3-(dimethylamino)propyl]-2-hydroxypropanamide	3
2-hydroxy-*N*-[3-(1*H*-imidazol-1-yl)propyl] propanamide	24

N-[2-(dimethylamino)ethyl]-2-hydroxypropanamide

Pale yellow oil. Yield: 88%. ^{1}H NMR, (400 MHz, DMSO) δ= 1.1 (3H, d, J=8Hz), 2.13 (6H, s), 2.28 (2H, t, J=8Hz), 3.15 (2H, m), 3.90 (1H, q, J=4Hz), 7.52 (1H, m) ppm.

N-[3-(dimethylamino)propyl]-2-hydroxypropanamide

Pale yellow oil. Yield: 90%. ^{1}H NMR, (400 MHz, DMSO) δ= 1.17 (3H, d, J=8Hz), 1.52 (2H, quint., J=8Hz), 2.09 (6H, s), 2.17 (2H, t, J=8Hz), 3.08 (2H, q, J=8Hz), 3.90 (1H, m), 5.41 (1H, m), 7.71 (1H, m) ppm.

2-hydroxy-N-[3-(1H-imidazol-1-yl)propyl] propanamide

Pale yellow oil. Yield:83 %. ^{1}H NMR, (400 MHz, DMSO) δ= 1.21 (3H, d, J=4Hz), 1.73 (1H, quint., J=8Hz), 3.05 (2H, m), 3.93 (4H, m), 6.87 (1H, s), 7.17 (1H, m), 7.62 (1H, m), 7.83 (1H, m) ppm. ^{13}C NMR, (400 MHz, DMSO) δ= 21.55, 31.32, 35.88, 44.09, 67.79, 119.80, 128.78, 138.25, 175.21 ppm.

General procedure for the alkylation of lactamides

The amide and the suitable alkyl halide (1.5 eq.) were solubilized in EtOH (2 mL) in a reaction vial. The resulting solution was heated to 70 °C for the reaction times reported below.

**Salt synthesized**	Reaction time (h)
**[N_{112}-Lac-4]I**	24
**[N_{112}-Lac12]I**	72
**[N_{113}-Lac-4]I**	24
**[N_{113}-Lac-6]I**	24
**[N_{113}-Lac-8]I**	72
**[N_{113}-Lac-10]I**	72
**[N_{113}-Lac-12]I**	72
**[Im_3-Lac-4]I**	72
**[Im_3-Lac-12]I**	168
**[N_{113}-Lac-4]Br**	72
**[N_{113}-Lac-12]Br**	72

Subsequently, the solvent was evaporated at reduced pressure. Then, the residue obtained was washed (3x 5 mL) with ethyl acetate or diethyl ether. In particular, diethyl ether was used to wash the following salts: **[N_{113}-Lac-8]I**, **[N_{113}-Lac-10]I**, **[N_{113}-Lac-12]I**, **[Im_3-Lac-4]I**, **[Im_3-Lac-12]I**.

[N_{112}-Lac-4]I

Yellow oil. Yield: 92%. FTIR (liquid film): 3333 (O-H stretch), 3250 (N-H stretch), 1654 (N-**C=O** stretch), 1121 (C-O stretch.) cm^{-1}. ^{1}H NMR, (400 MHz, DMSO) δ= 0.92 (3H, t, J=8Hz), 1.22 (3H, d, J=8Hz), 1.28 (2H, m), 1.64 (2H, m), 3.04 (6H, s), 3.31 (6H, m), 3.98 (1H, m), 5.67 (1H, m), 8.12 (1H, m) ppm. ^{13}C NMR (400 MHz, DMSO) δ= 13.9, 19.6, 21.4, 24.1, 31.2, 33.1, 51.1, 61.5, 63.5, 67.7, 176.2 ppm.

[N_{112}-Lac-12]I

Yellow oil. Yield: 93%. FTIR (liquid film): 3334 (O-H stretch), 1653 (N-**C=O** stretch), 1125 (C-O stretch) cm^{-1}. ^1H NMR, (400 MHz, DMSO) δ = 0.84 (3H, m), 1.21 (4H, m), 1.24 (18H, m), 1.65 (2H, m), 3.03 (6H, s), 3.31 (4H, m), 3.99 (1H, m), 5.66 (1H, m), 8.13 (1H, m) ppm. ^{13}C NMR (400 MHz, DMSO) δ= 14.4, 21.4, 22.1, 22.5, 26.2, 29.0, 29.2, 29.3, 29.4, 29.5, 31.2, 31.8, 33.1, 51.1, 62.1, 64.4, 67.7, 175.6 ppm.

[N_{113}-Lac-4]I

Yellow solid. Yield: 95%. FTIR (nujol mull): 3371 (O-H stretch), 1652 (N-**C=O** stretch), 1124 (C-O stretch) cm^{-1}. ^1H NMR, (400 MHz, DMSO) δ = 0.92 (3H, m), 1.22 (3H, d, J=8Hz), 1.30 (2H, q, J=8 Hz), 1.61 (2H, m), 1.82 (2H, m), 2.99 (6H, s), 3.12 (2H, q, J=8 Hz), 3.23 (3H, q, J=8 Hz), 3.96 (1H,m), 5.47 (1H, d, J=8 Hz), 7.88 (1H, t, J=8 Hz) ppm. ^{13}C NMR (400 MHz, DMSO) δ= 13.9, 19.6, 21.52, 23.0, 24.1, 35.7, 50.1, 61.4, 63.3, 68.2, 175.3 ppm.

[N_{113}-Lac-6]I

Orange oil. Yield: 97%. %. FTIR (liquid film): 3382 (O-H stretch), 1655 (N-**C=O** stretch.), 1122 (C-O stretch.) cm^{-1}. ^1H NMR, (400 MHz, $CDCl_3$) δ = 0.88 (3H, m), 1.24 (1H, m), 1.34 (4H, m), 1.38 (4H, m), 1.74 (2H, m), 2.09 (2H, m), 3.27 (6H, s), 3.37 (3H, m), 3.56 (1H, m), 3.71 (2H, m), 4.09 (1H, q, J= 4Hz), 4.31 (1H, m), 7.61 (1H, m) ppm. ^{13}C NMR (400 MHz, DMSO) δ= 13.9, 20.5, 22.4, 22.8, 23.1, 25.9, 31.2, 35.7, 51.4, 62.8, 64.9, 68.1, 176.6.

[N_{113}-Lac-8]I

Orange oil. Yield: 98%. FTIR (liquid film): 3372 (O-H stretch), 1653 (N-**C=O** stretch), 1123 (C-O stretch.) cm^{-1}. 1H NMR, (400 MHz, CDCl3) δ = 0.88 (3H, m), 1.36 (12H, m), 1.73 (2H, m), 2.09 (4H, m), 3.38 (8H, m), 3.58 (1H, m), 3.73 (2H, m), 4.34 (1H, q, J=12 Hz), 4.62 (1H, m), 7.63 (1H, s). ^{13}C NMR (400 MHz, DMSO) δ= 14.1, 20.5, 22.6, 22.9, 23.1, 26.3, 29.0, 29.1, 31.6, 35.6, 51.4, 62.9, 64.9, 68.1, 176.2 ppm.

[N_{113}-Lac-10]I

Orange oil. Yield: 83%. FTIR (liquid film): 3361 (O-H stretch), 1653 (N-**C=O** stretch), 1123 (C-O stretch.) cm^{-1}. ^1H NMR, (400 MHz, $CDCl_3$) δ = 0.88 (3H,m), 1.26 (12H, m), 1.37 (3H, m), 1.41 (3H, d, J=8 Hz), 1.74 (2H, m), 2.11 (2H, m), 3.29 (6H, s), 3.38 (3H, m), 3.61 (1H, m), 3.77 (2H, m), 4.36 (1H, m), 7.61 (1H, m) ppm. ^{13}C NMR (400 MHz, DMSO) δ= 13.0, 19.8, 22.2, 22.3, 22.7, 26.0, 28.0, 29.0, 29.2, 28.2, 31.6, 35.5, 50.1, 61.8, 64.3, 67.7, 176.6 ppm.

[N_{113}-Lac-12]I

Orange oil. Yield: 92%. FTIR (liquid film): 3361 (O-H stretch), 1653 (N-**C=O** stretch), 1123 (C-O stretch) cm^{-1}. ^{1}H NMR, (400 MHz, $CDCl_3$) δ = 0.88 (3H, m), 1.26 (14H, s), 1.36 (3H, m), 1.44 (3H, d, J=8Hz), 1.74 (2H, m), 2.13 (4H, m), 3.28 (6H, s), 3.37 (2H, m), 3.60 (1H, m), 3.76 (2H, m), 4.35 (1H, m), 7.61 (1H, m) ppm. ^{13}C NMR (400 MHz, DMSO) δ=14.1, 20.5, 22.7, 22.9, 23.1, 26.3, 29.2, 29.3 (2C, overlapped), 29.4, 29.5, 29.6, 31.9, 35.6, 51.4, 63.2, 64.5, 68.9, 176.5 ppm.

[Im_3-Lac-4]I

Orange oil. Yield: 99%. FTIR (liquid film): 3395(O-H stretch), 1653 (N-**C=O** stretch), 1163 (C-O stretch.) cm^{-1}. ^{1}H NMR, (400 MHz, DMSO) δ = 0.89 (3H, t, J=8Hz), 1.23 (4H, m), 1.77 (2H, m), 1.95 (2H, quin., J=8Hz), 3.06 (2H, m), 3.96 (1H, q, J=8Hz), 4.14 (3H, m), 5.48 (1H, s), 7.80 (2H, d, J=8Hz), 7.89 (1H, m), 9.19 (1H, s) ppm. ^{13}C NMR (400 MHz, DMSO) δ= 13.78, 19.3, 21.6, 30.1, 31.7, 35.3, 47.2, 49.1, 67.8, 122.9, 136.6, 175.3 ppm.

[Im_3-Lac-12]I

Orange oil. Yield: 97%. FTIR (liquid film): 3382 (O-H stretch), 1653 (N-**C=O** stretch), 1560 (amide II), 1163 (C-O stretch.) cm^{-1}. ^{1}H NMR, (400 MHz, DMSO) δ = 0.89 (3H, m), 1.19 (2H, d, J=4Hz), 1.25 (2H, q, J=8Hz), 1.77 (2H, quin, J=8Hz), 1.95 (2H, quin, J=8Hz), 3.06 (2H, m), 3.45 (2H, m), 3.96 (1H, q, J=4Hz), 4.14 (3H, m), 5.48 (1H, s), 7.80 (2H,m), 9.19 (1H, s) ppm. ^{13}C NMR (400 MHz, DMSO) δ=13.0, 19.9, 22.3, 25.9, 28.7, 29.0, 29.1, 29.2, 29.3, 29.67, 29.8, 31.7, 35.0, 49.7, 68.4, 122.4, 135.3, 136.1,176.9 ppm.

[N_{113}-Lac-4]Br

Yellow solid. Yield: 91%. FTIR (nujol film): 3301 (O-H stretch), 1654 (N-**C=O** stretch) cm^{-1}. ^{1}H NMR (400 MHz, DMSO)= 0.92 (3H, t, J=8Hz), 1.19 (3H, d, J=8Hz), 1.29 (2H, sext, J=8Hz), 1.62 (2H, m), 1.82 (2H, m), 3.01 (6H, s), 3.13 (2H, m), 3.24 (4H, m), 3.96 (1H, m), 5.49 (1H, d, J=4Hz), 7.90 (1H, m). ^{13}C NMR (400 MHz, DMSO) = 14.2, 19.9, 21.3, 23.1, 24.5, 35.7, 50.5, 61.4, 63.1, 68.0, 175.3 ppm

[N_{113}-Lac-12]Br

Yellow oil. Yield: 70%. FTIR (liquid film): 3361 (O-H stretch), 1653 (N-**C=O** stretch), 1162 (C-O stretch) cm^{-1}. ^{1}H NMR (400 MHz, DMSO)= 0.85 (3H, m), 1.24 (19H, m), 1.59 (2H, m), 1.81 (2H, m), 2.75 (2H, m), 2.98 (6H, s), 3.12 (2H, m), 3.21 (4H, m), 3.96 (1H, m), 5.49 (1H, m), 7.91 (1H, m) ppm. ^{13}C NMR (400 MHz, DMSO) = 14.4, 21.52, 22.1, 22.5, 26.2, 26.3, 29.0, 29.2, 29.3, 29.4, 31.7, 35.7, 42.7, 50.6, 54.7, 60.9, 63.7, 68.2, 176.2 ppm.

Differential Scanning Calorimetry. DSC measurements were carried out on a Q20 instrument calibrated using indium as standard. Samples for a typical measurement were sealed in aluminum pans with hermetic lids. Unless otherwise stated, heating and cooling rates were 10 °C/min. For each run, the temperature was ramped from 25 to 60 °C, then the sample was cooled down to -40 °C under nitrogen atmosphere. For **[N_{113}-Lac_4][I]** and **[N_{113}-Lac_4][Br]**, the temperature range was comprised between 0 °C and 120 °C and between 0 °C and 150 °C, respectively. For each sample, at least three heat-cool cycles were carried out.

Thermogravimetric analysis. The temperatures of decomposition, as well as the mass loss, were measured using a TA instrument TGA Q5000 thermogravimetric analyzer, at a heating rate of 5 °C min−1 under nitrogen flow.
The maximum values of the DTGA curves of each thermogram were used as a measure of the decomposition temperature. The mass loss was calculated from the area of the peaks exhibited by the DTG curves.

Cell Culture. The IMR-90 (ATCC CCL-186™) fibroblasts isolated from normal lung tissue derived from a 16-week-old female were cultured in Dulbecco's modified Eagle medium (DMEM) supplemented with glutamine, with 10% heat-inactivated foetal bovine serum (FBS), 100 U/mL penicillin and 100 mg/L streptomycin, as previously reported. [1]The cells were grown as an adherent monolayer culture in standard conditions (95% humidity, 5% CO2, 37°C).

Cytotoxicity Assay. Twenty-four hours before adding the tested ILs, the cells (20×10^3 cells/well) were seeded onto 96-well plates (Sarstedt). The next day, solutions of ILs were prepared in the culture medium (in a concentration range from 100 to 0.0064 μM) and then added to the cells. Stock solutions of salts (10^{-3} M), dissolved in DMSO were used. The cytotoxicity assay was performed after 24 h by exchanging the medium with testes ILs with fresh DMEM (100 μL) containing 20 μL of thiazolyl blue tetrazolium bromide (5 mg mL^{-1}), and culturing for 2 h at 37 °C in a CO2 incubator. After removing the medium-containing MTT and three washes with PBS, 100 μL of warm DMSO was added to each well to dissolve formazan. Samples were shaken for 10 min, at room temperature in the dark, and the absorbance was recorded at 570 nm using a microplate reader (Spark 20M Tecan Trading AG, Switzerland). For calculation of the IC_{50} values, the absorbance of cells incubated with tested ILs was compared to the absorbance of untreated cells. IC_{50} values along with standard deviations (confidence level 0.95) were determined using the GraphPad Prism 8 software. Each experiment was repeated three times in triplicate (for each IL).

Morphological evaluation. Cells were seeded (20 × 10^3 cells/well) onto 96-well plates and after 24 h treated with different salts concentrations. Stock solutions of salts (10^{-2} M), dissolved in DMSO were used. After 24 h of treatment cells observed under a inverted microscope (Carl Zeiss AG, Oberkochen, Germany). Magnification 200X.

Table S1. Optimisation of reaction condition for the aminolysis of PLA in the presence of *N,N*-dimethylpropylenediamine.

**Reaction Conditions**	Conversion (%)	Yield (%)
40 °C, 1h, 1.5 eq.	18	17
40 °C, 3h, 1.5 eq.	45	45
40 °C, 6h, 1.5 eq.	62	60
40 °C, 3h, 2 eq.	52	52
40 °C, 1h, 1.5 eq.	18	17
70 °C, 1h, 1.5 eq.	75	73
100 °C, 1h, 1.5 eq.	99	81
70 °C, 1h, 1.5 eq.	75	73
70 °C, 3h, 1.5 eq.	92	90

Table S2. Conversion, yield and selectivity values for the aminolysis of PLA in the presence of different nucleophiles. Results are evaluated on the ground of the holistic approach to Green Chemistry (Flag).[2]

**Reaction Conditions**	**Conversion (%)**	**Flag**[2]	**Yield (%)**	**Flag**[2]	**Selectivity (%)**	**Flag**[2]
70 °C, 3h, 1.5 eq. DMPDA	92	Green	90	Green	98	Green
70 °C, 3h, 1.5 eq. DMEDA	90	Green	88	Yellow	98	Green
70 °C, 3h, 1.5 eq. API	41	Red	18	Red	44	Red
70 °C, 24h, 1.5 eq. API	89	Green	83	Yellow	93	Green

Table S3. Optimisation of reaction condition for the alkylation of **[N_{113}-Lac]** in the presence of butyl or dodecyl iodide at 70 °C, for 24h.

Solvent	Alkyl iodide	Eq	Yield (%)
-	C_4H_9I	1.5	-
	$C_{12}H_{25}I$	1.5	-
EtOH	C_4H_9I	1.0	10
EtOH	C_4H_9I	1.2	12
EtOH	C_4H_9I	1.5	95
EtOH	$C_{12}H_{25}I$	1.0	-
EtOH	$C_{12}H_{25}I$	1.2	-
EtOH	$C_{12}H_{25}I$	1.5	93

Table S4. Glass transition (T_g) and melting temperature (T_m), thermodynamic parameters determined by DSC measurements relevant to salt synthesised.

**Salt**	**Heating** T_g **(°C) or** T_m **(°C)**	**Cooling** T_g **(°C)**
**[N_{112}-Lac-4][I]**	-8.16	-
**[N_{112}-Lac-12][I]**	-	-
**[N_{113}-Lac-4][I]**	99.74	-
**[N_{113}-Lac-6][I]**	-	-
**[N_{113}-Lac-8][I]**	-	-
**[N_{113}-Lac-10][I]**	-	-
**[N_{113}-Lac-12][I]**	-	-
**[Im_3-Lac-4][I]**	-	-
**[Im_3-Lac-12][I]**	-24.91	-11.98
**[N_{113}-Lac-4][Br]**	59.77 (I); 120.45 (I, II)	-
**[N_{113}-Lac-12] [Br]**	-	-

Table S5. IC_{50} values relevant to the ILs obtained.

	**IC_{50} µM ± SD**
**[N_{113}-Lac-4]I**	68.85±7.01
**[N_{113}-Lac-6]I**	84.99 ±18.21
**[N_{113}-Lac-8]I**	13.66 ±2.24
**[N_{113}-Lac-10]I**	11.62 ±0.99
**[N_{113}-Lac-12]I**	6.95±2.65
**[N_{113}-Lac-4]Br**	94.43±21.66
**[N_{113}-Lac-12]Br**	14.79±2.32
**[N_{112}-Lac-4]I**	82.18±5.21
**[N_{112}-Lac-12]I**	12.24±1.53
**[Im_3-Lac-4]I**	61.6±3.57
**[Im_3-Lac-12]I**	6.58±0.73

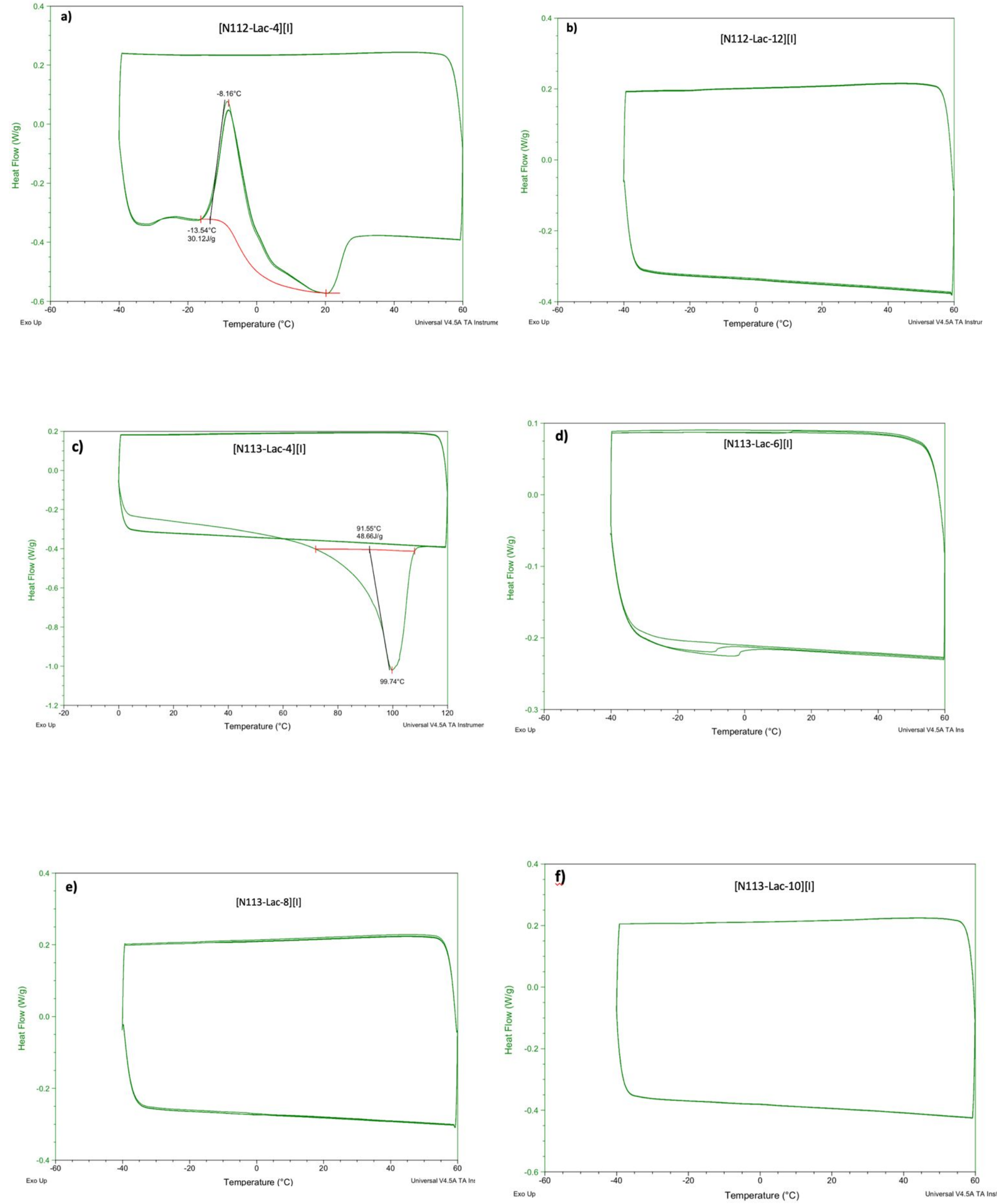

a)
[N112-Lac-4][I]
-8.16°C
-13.54°C
30.12J/g
Heat Flow (W/g)
Temperature (°C)
Exo Up
Universal V4.5A TA Instrume
b)
[N112-Lac-12][I]
c)
[N113-Lac-4][I]
91.55°C
48.66J/g
99.74°C
d)
[N113-Lac-6][I]
e)
[N113-Lac-8][I]
f)
[N113-Lac-10][I]

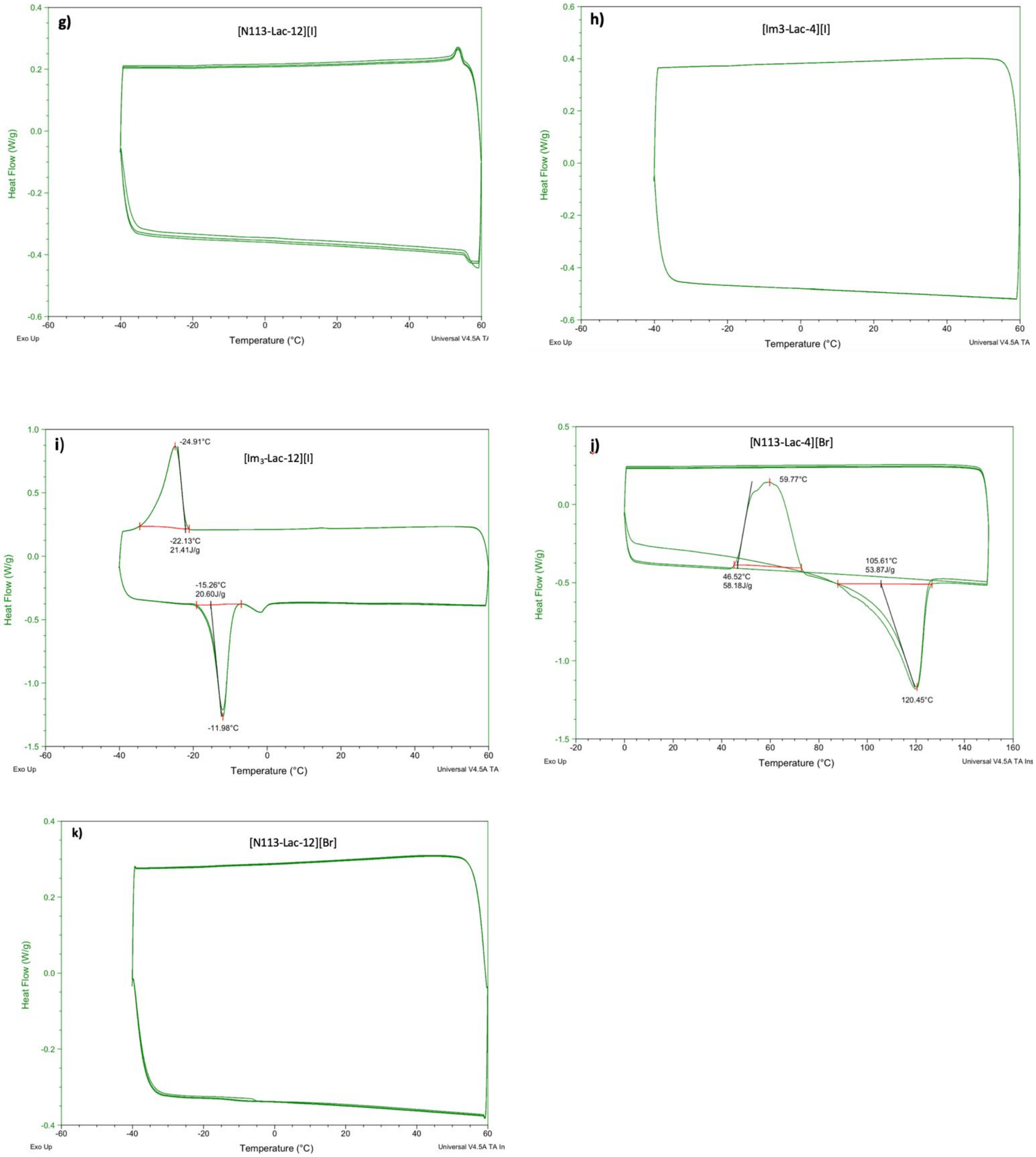

Figure S1. DSC traces of a) **[N_{112}-Lac-4][I], b) [N_{112}-Lac-12][I],** c) **[N_{113}-Lac-4][I],** d) **[N_{113}-Lac-6][I],** e) **[N_{113}-Lac-8][I],** f) **[N_{113}-Lac-10][I],** g) **[N_{113}-Lac-12][I],** h) **[Im_3-Lac-4][I],** i) **[Im_3-Lac-12][I],** j) **[N_{113}-Lac-4][Br] and** k) **[N_{113}-Lac-12][I].**

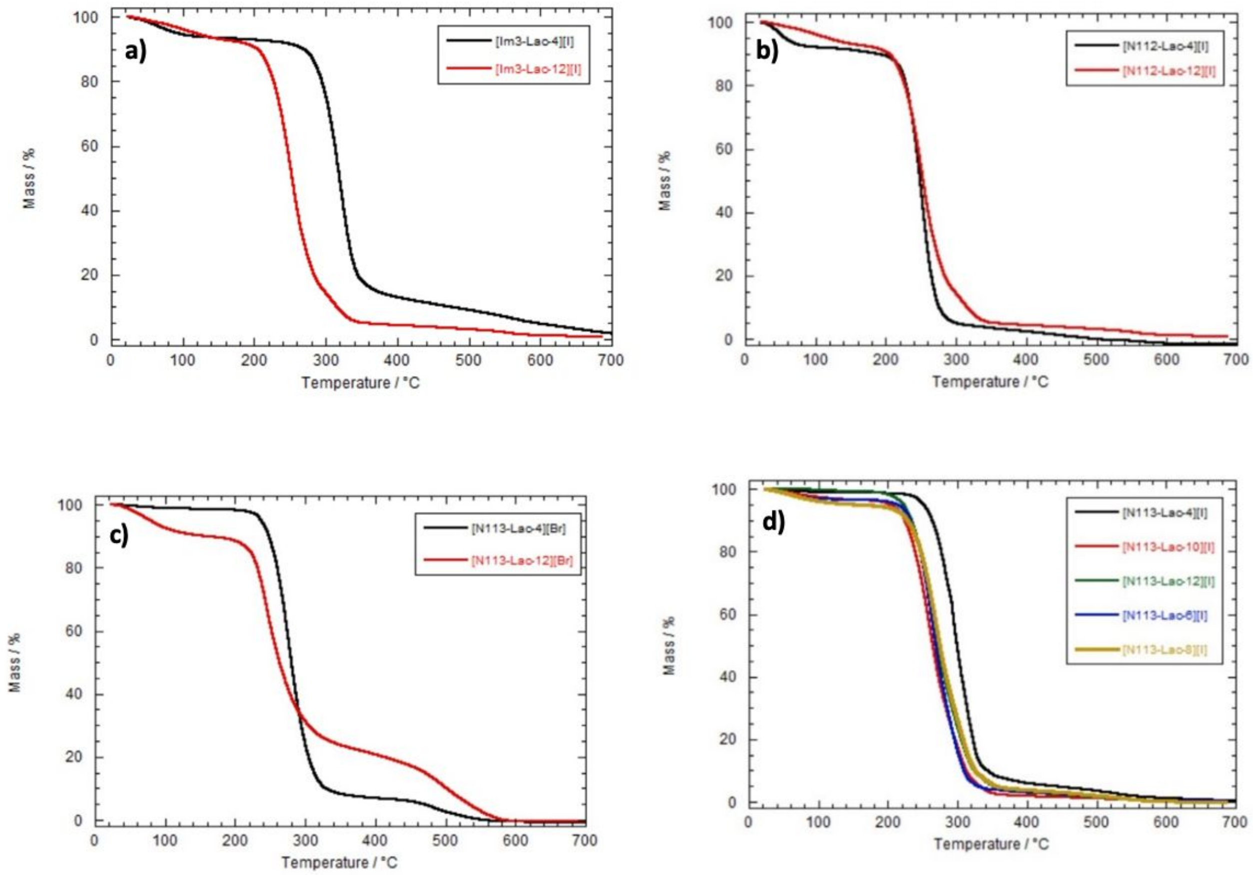

Figure S2. Thermogravimetric curves of a) **[Im$_3$-Lac-4][I]** and **[Im$_3$-Lac-12][I],** b) **[N$_{112}$-Lac-4][I] and [N$_{112}$-Lac-12][I]**, c) **[N$_{113}$-Lac-4][Br]** and **[N$_{113}$-Lac-12][Br],** d) **[N$_{113}$-Lac-4][I], [N$_{113}$-Lac-6][I], [N$_{113}$-Lac-8][I], [N$_{113}$-Lac-10][I]** and **[N$_{113}$-Lac-12][I].**

a)

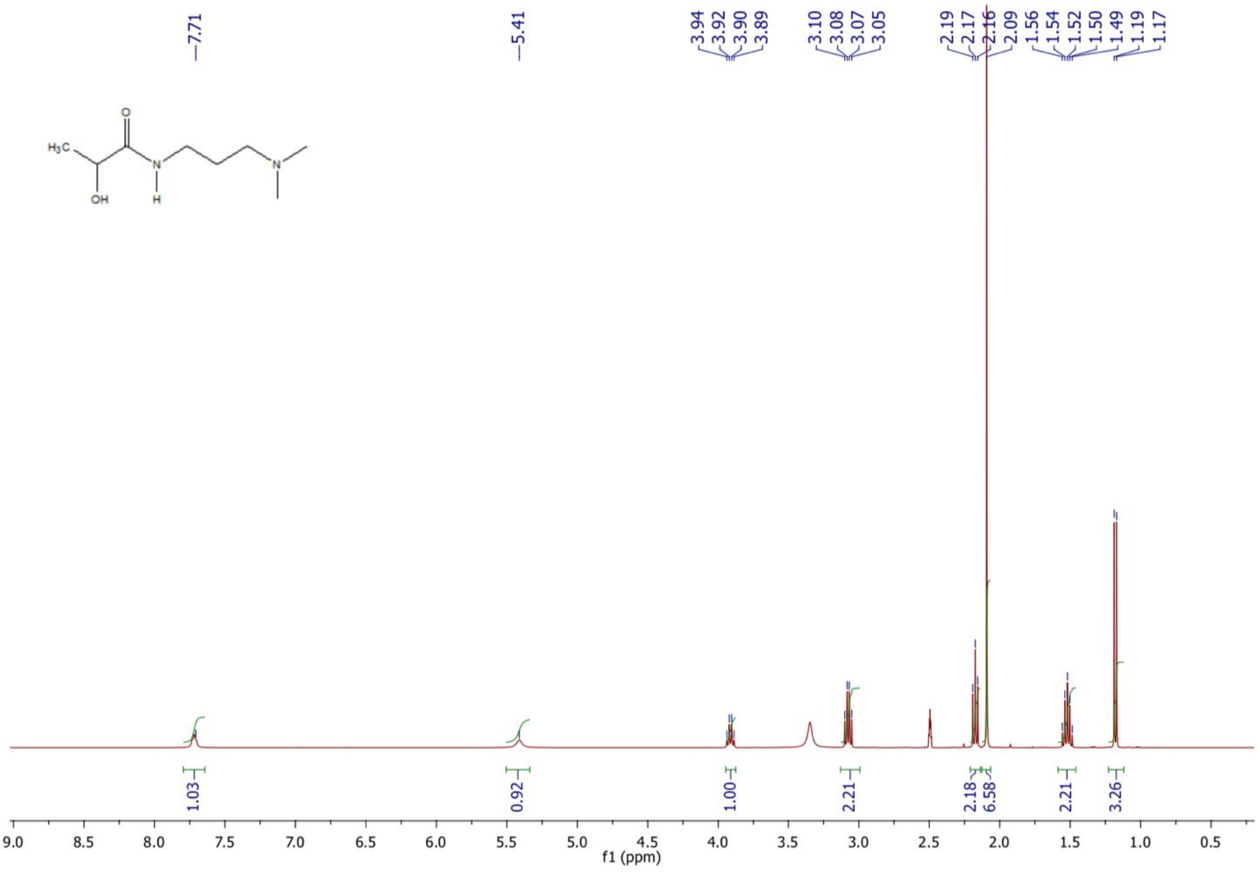

7.71
5.41
3.94 3.92 3.90 3.89
3.10 3.08 3.07 3.05
2.19 2.17 2.16 2.09
1.56 1.54 1.52 1.50 1.49
1.19 1.17
H3C
O
N
H
OH
N
1.03
0.92
1.00
2.21
2.18
6.58
2.21
3.26
9.0 8.5 8.0 7.5 7.0 6.5 6.0 5.5 5.0 4.5 4.0 3.5 3.0 2.5 2.0 1.5 1.0 0.5
f1 (ppm)

b)

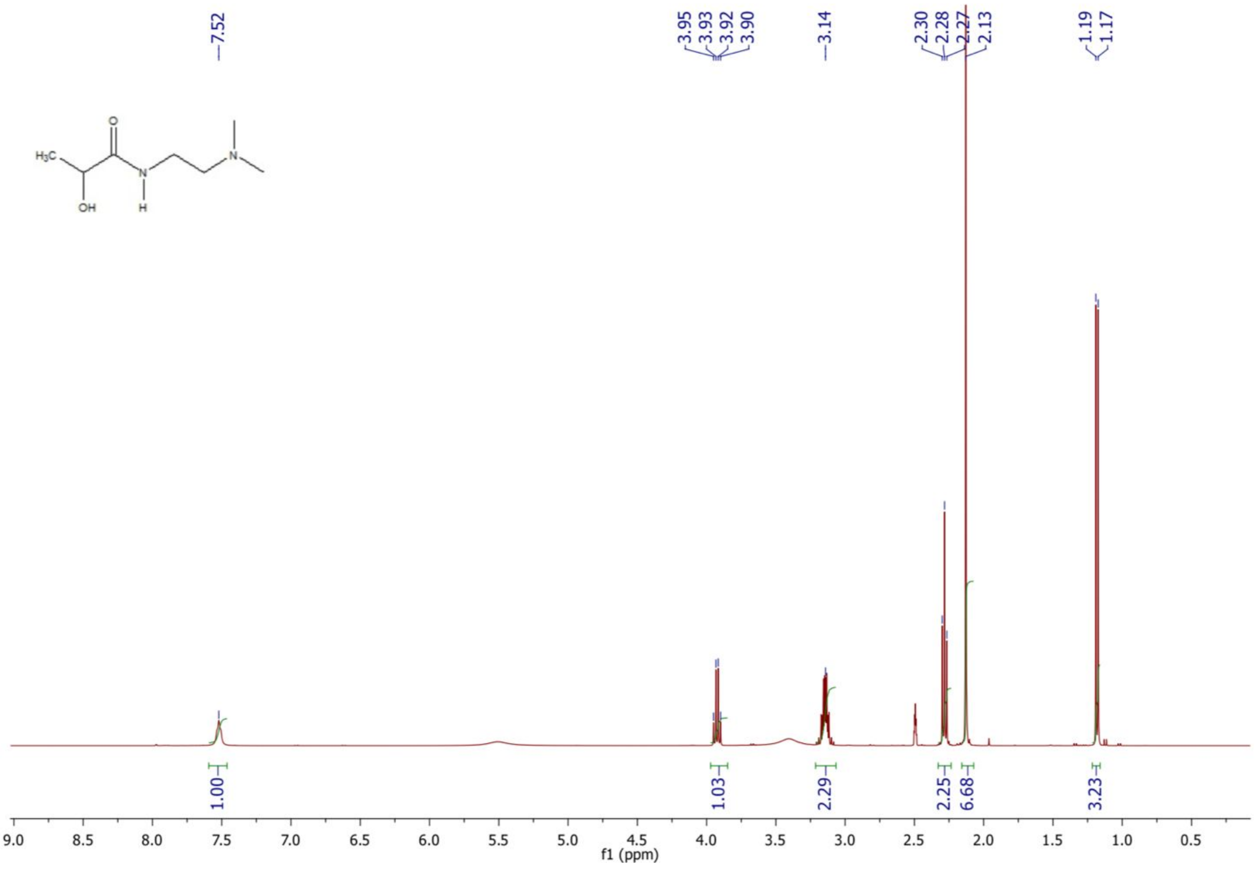

7.52
3.95 3.93 3.92 3.90
3.14
2.30 2.28 2.27 2.13
1.19 1.17
H3C
O
N
H
OH
N
1.00
1.03
2.29
2.25
6.68
3.23
9.0 8.5 8.0 7.5 7.0 6.5 6.0 5.5 5.0 4.5 4.0 3.5 3.0 2.5 2.0 1.5 1.0 0.5
f1 (ppm)

c)

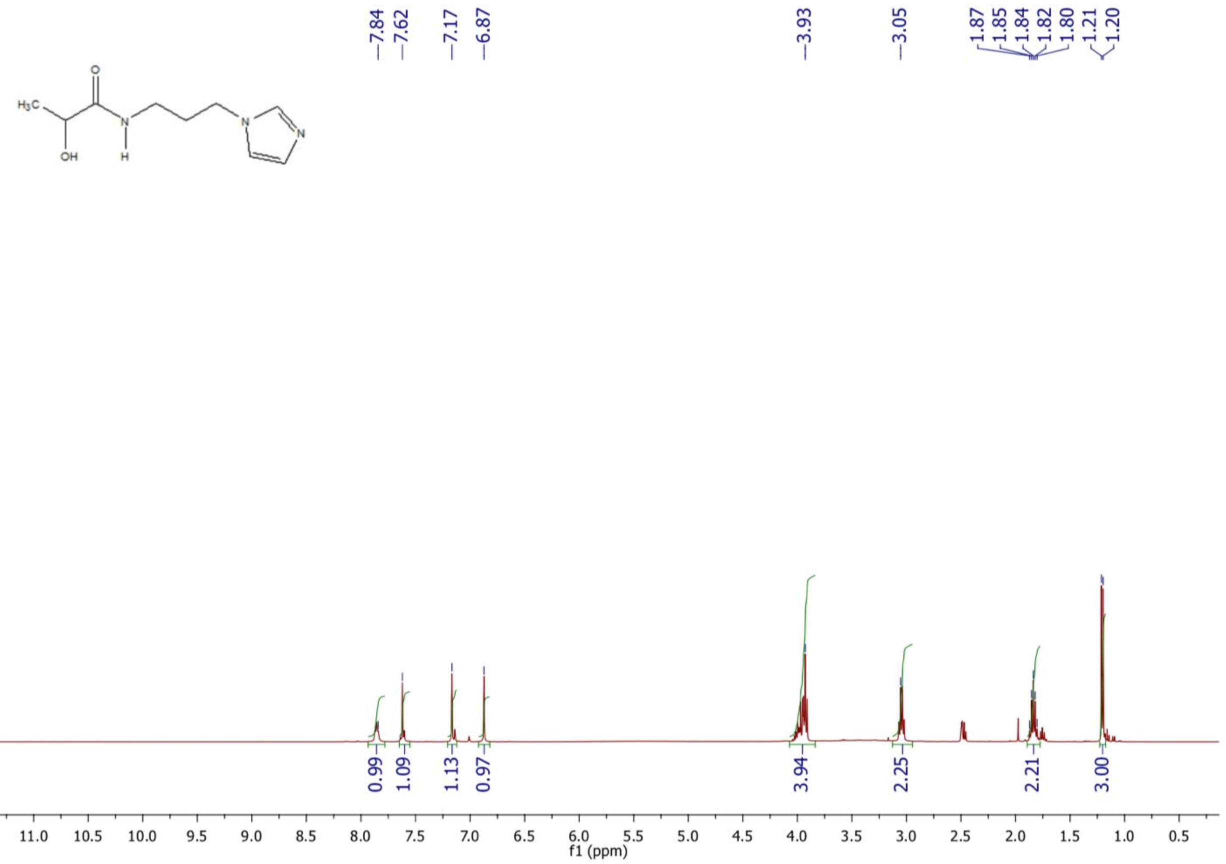
H3C
O
N
H
OH
N
N
7.84
7.62
7.17
6.87
3.93
3.05
1.87
1.85
1.84
1.82
1.80
1.21
1.20
0.99
1.09
1.13
0.97
3.94
2.25
2.21
3.00
11.0 10.5 10.0 9.5 9.0 8.5 8.0 7.5 7.0 6.5 6.0 5.5 5.0 4.5 4.0 3.5 3.0 2.5 2.0 1.5 1.0 0.5
f1 (ppm)

d)

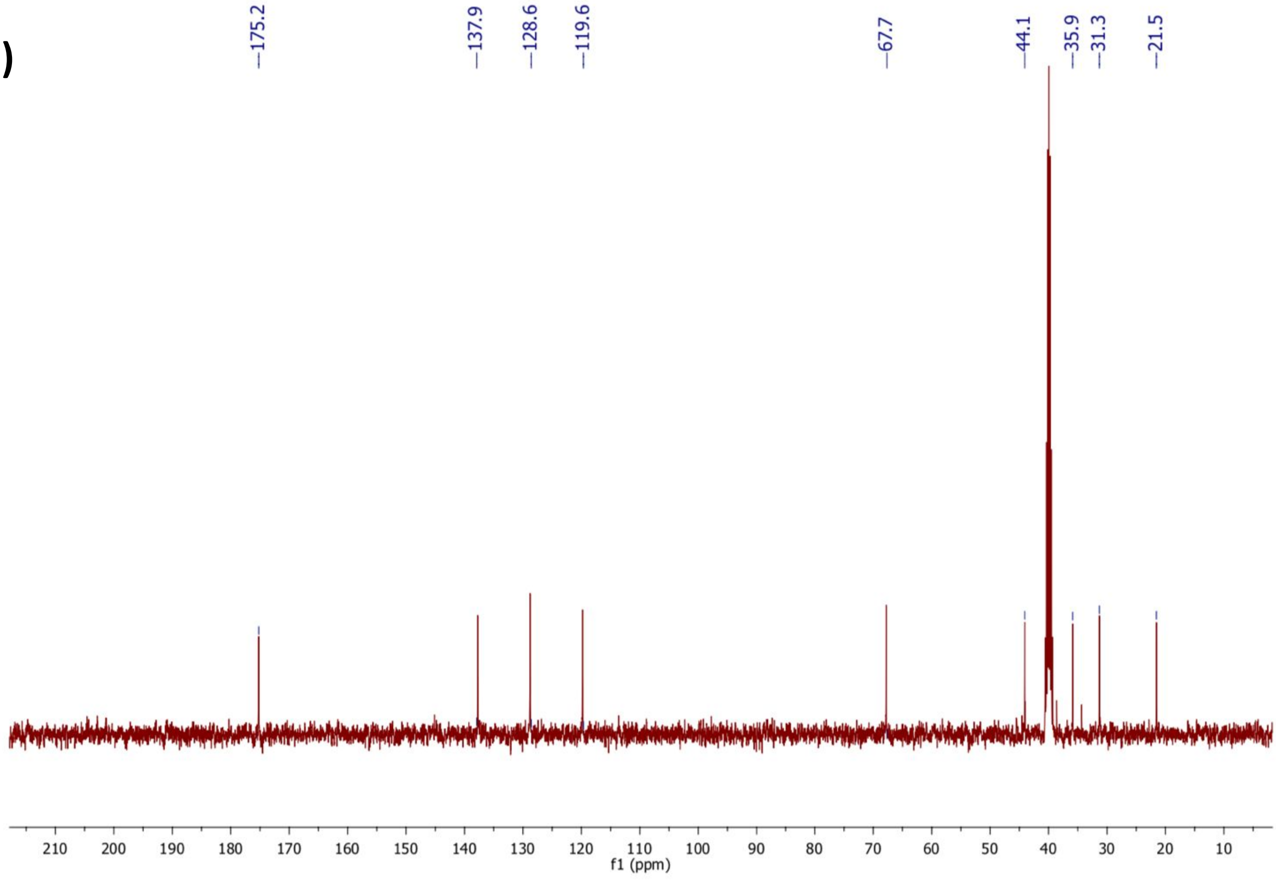
175.2
137.9
128.6
119.6
67.7
44.1
35.9
31.3
21.5
210 200 190 180 170 160 150 140 130 120 110 100 90 80 70 60 50 40 30 20 10
f1 (ppm)

e)

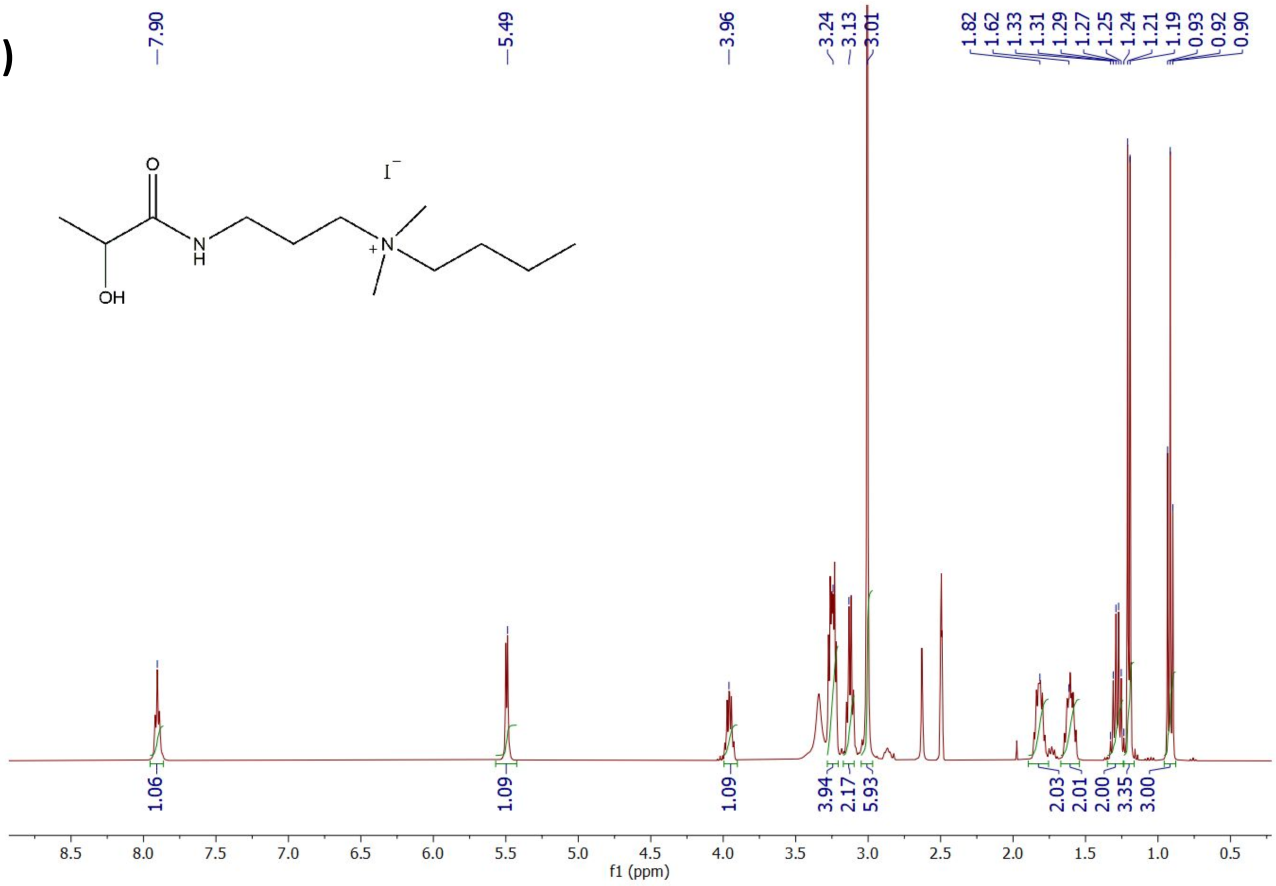
7.90
5.49
3.96
3.24
3.13
3.01
1.82
1.62
1.33
1.31
1.29
1.27
1.25
1.24
1.21
1.19
0.93
0.92
0.90
OH
H
N
+N
I−
1.06
1.09
1.09
3.94
2.17
5.93
2.03
2.01
2.00
3.35
3.00
8.5 8.0 7.5 7.0 6.5 6.0 5.5 5.0 4.5 4.0 3.5 3.0 2.5 2.0 1.5 1.0 0.5
f1 (ppm)

f)

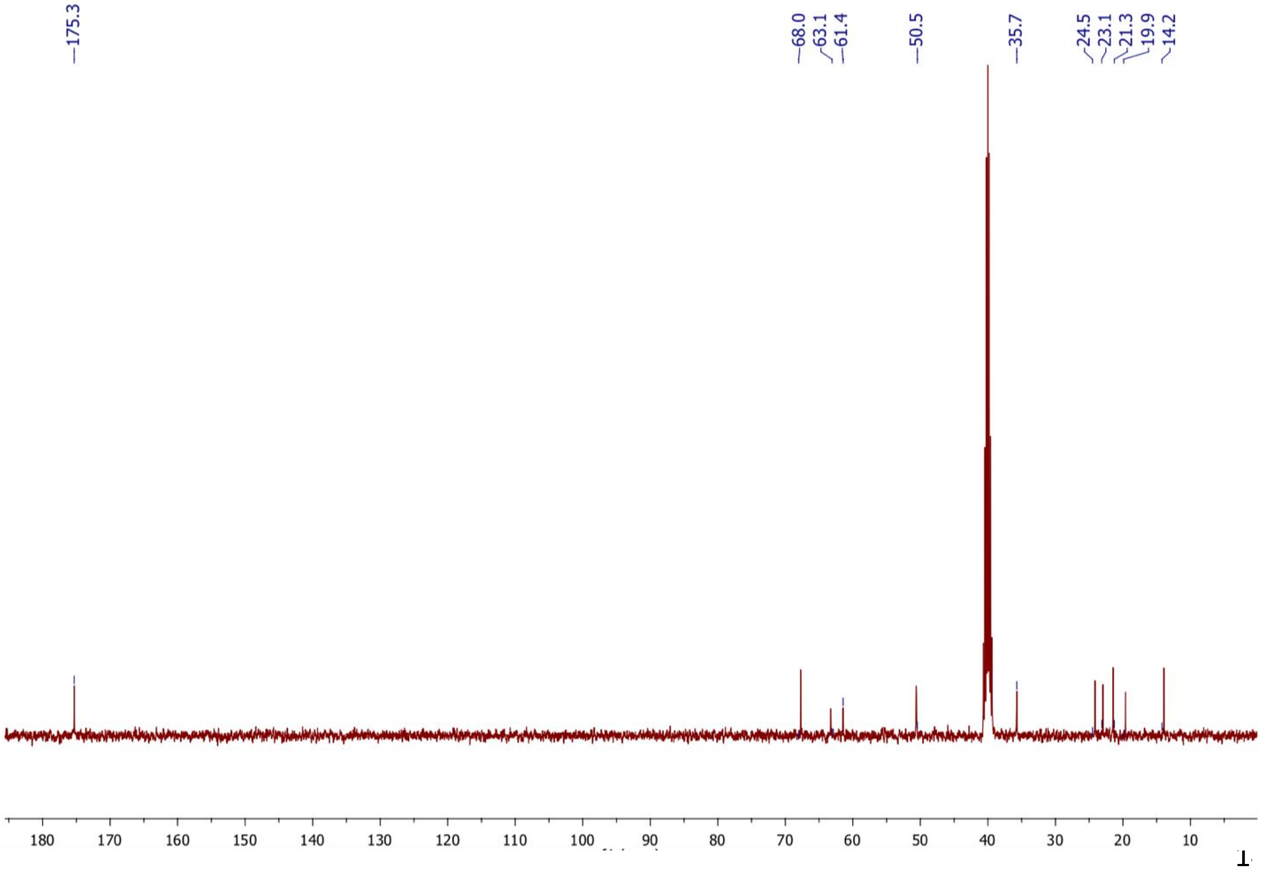
175.3
68.0
63.1
61.4
50.5
35.7
24.5
23.1
21.3
19.9
14.2
180 170 160 150 140 130 120 110 100 90 80 70 60 50 40 30 20 10

g)

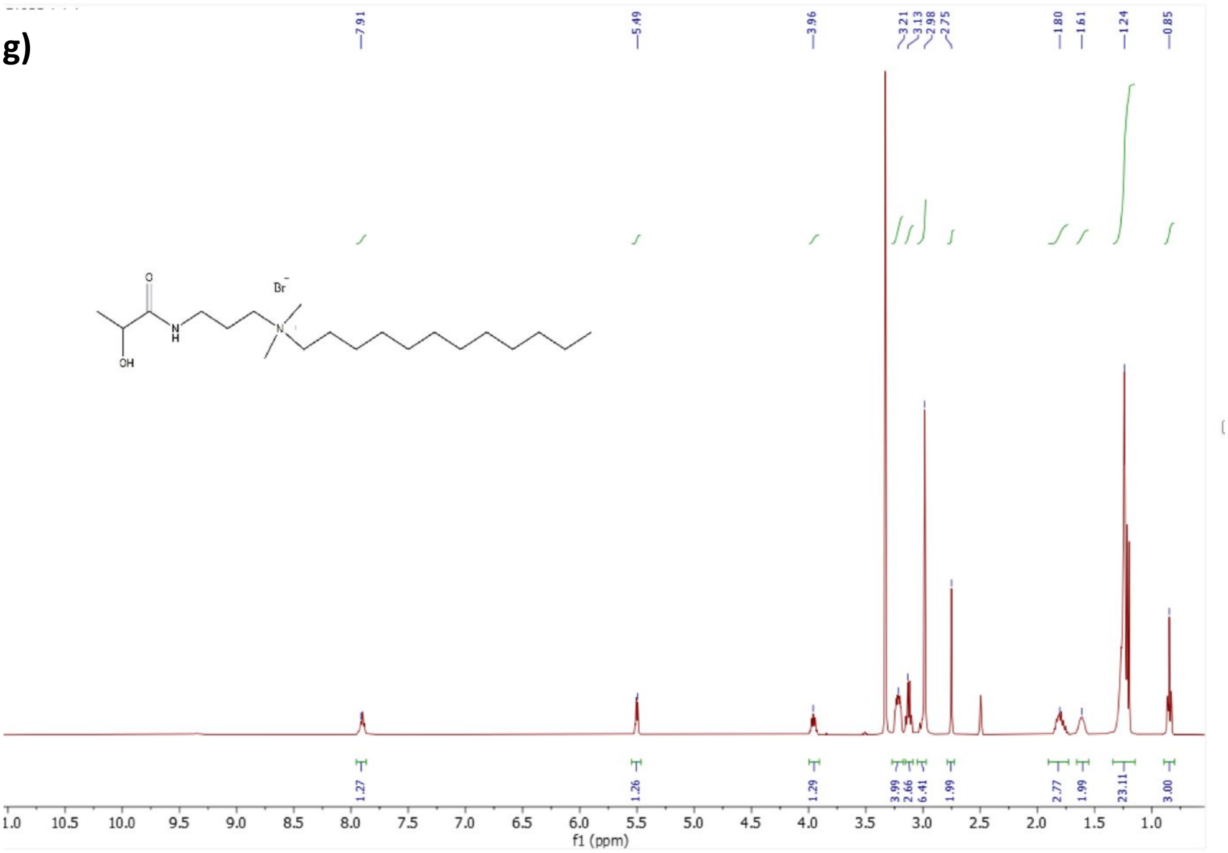
7.91
5.49
3.96
3.21
3.13
2.98
2.75
1.80
1.61
1.24
0.85
1.27
1.26
1.29
3.99
2.66
6.41
1.99
2.77
1.99
23.11
3.00
f1 (ppm)

h)

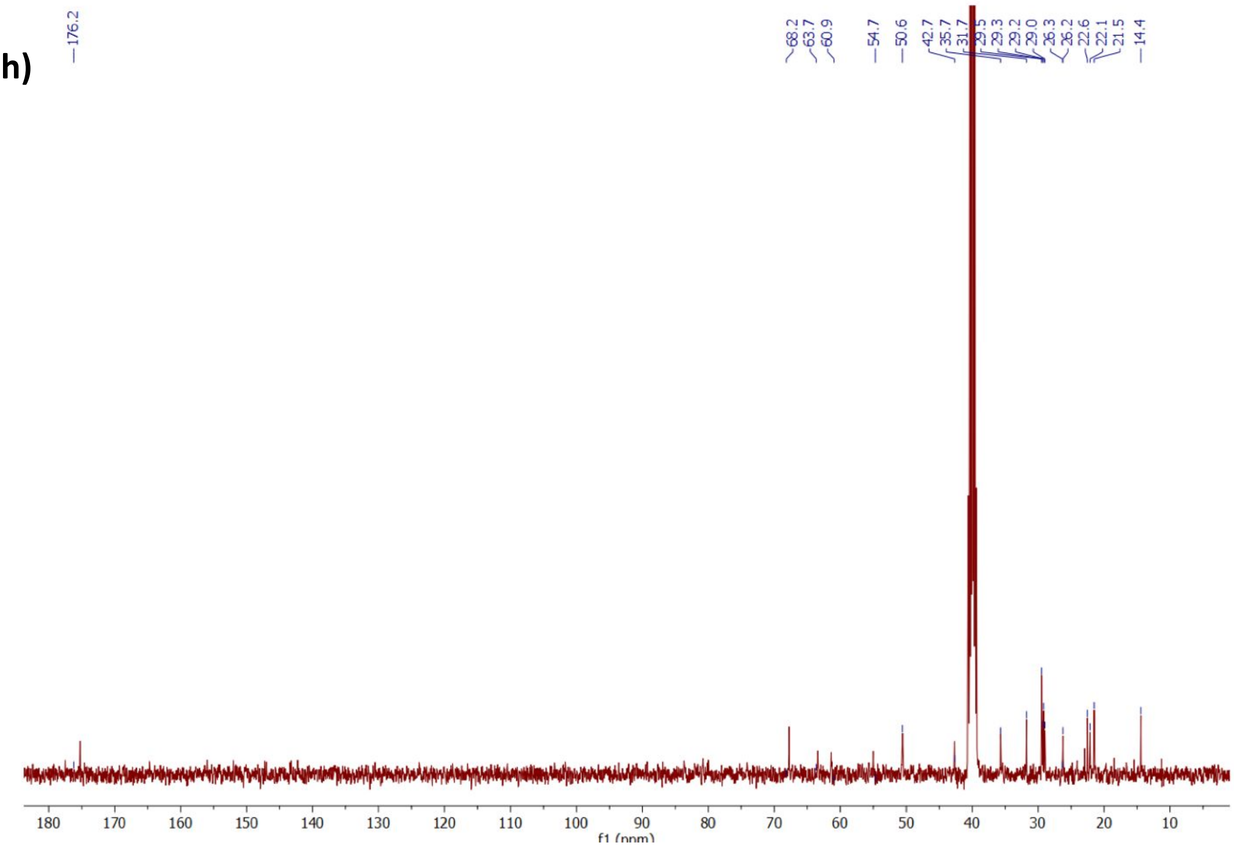
176.2
68.2
63.7
60.9
54.7
50.6
42.7
35.7
31.7
29.5
29.3
29.2
29.0
26.3
26.2
22.6
22.1
21.5
14.4

i)

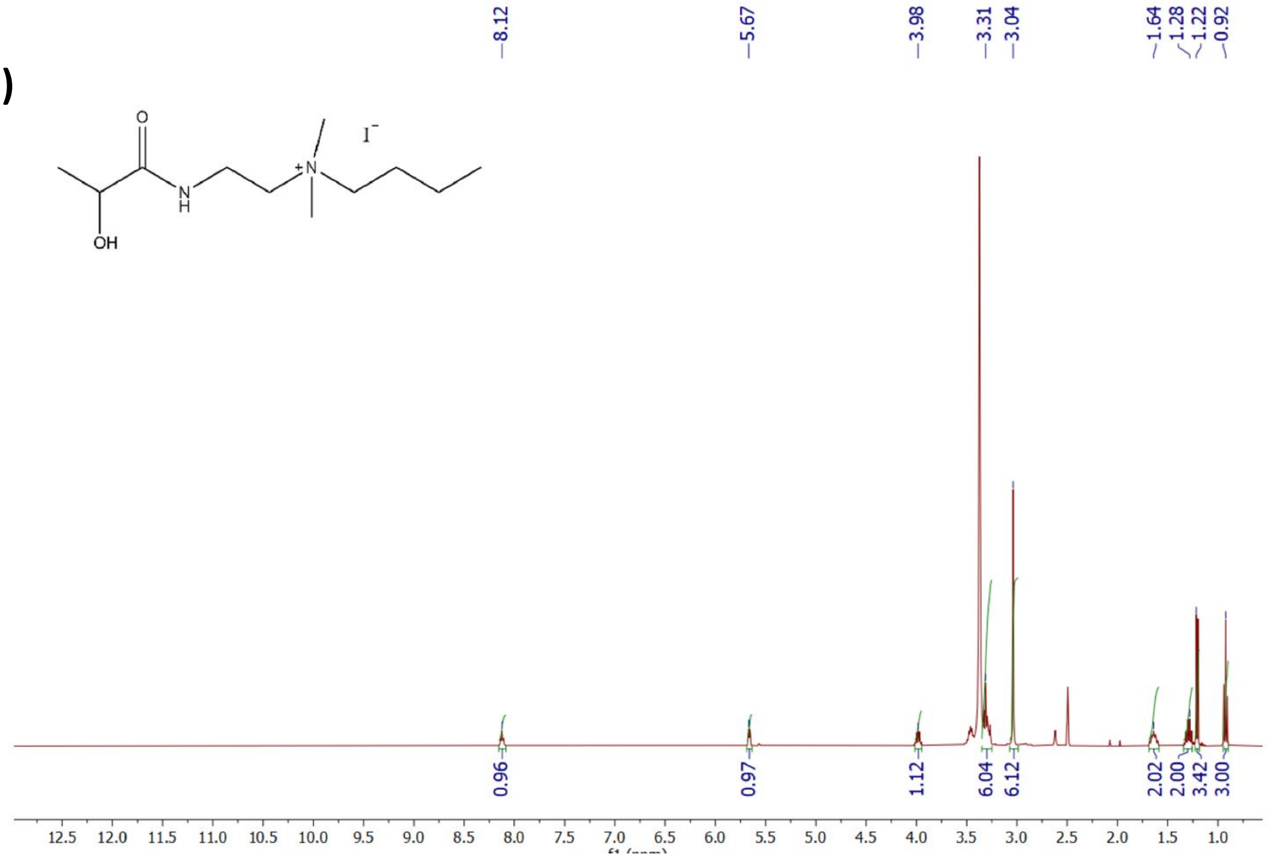
8.12
5.67
3.98
3.31
3.04
1.64
1.28
1.22
0.92
O
N
H
OH
+N
I⁻
0.96
0.97
1.12
6.04
6.12
2.02
2.00
3.42
3.00
12.5 12.0 11.5 11.0 10.5 10.0 9.5 9.0 8.5 8.0 7.5 7.0 6.5 6.0 5.5 5.0 4.5 4.0 3.5 3.0 2.5 2.0 1.5 1.0
f1 (ppm)

j)

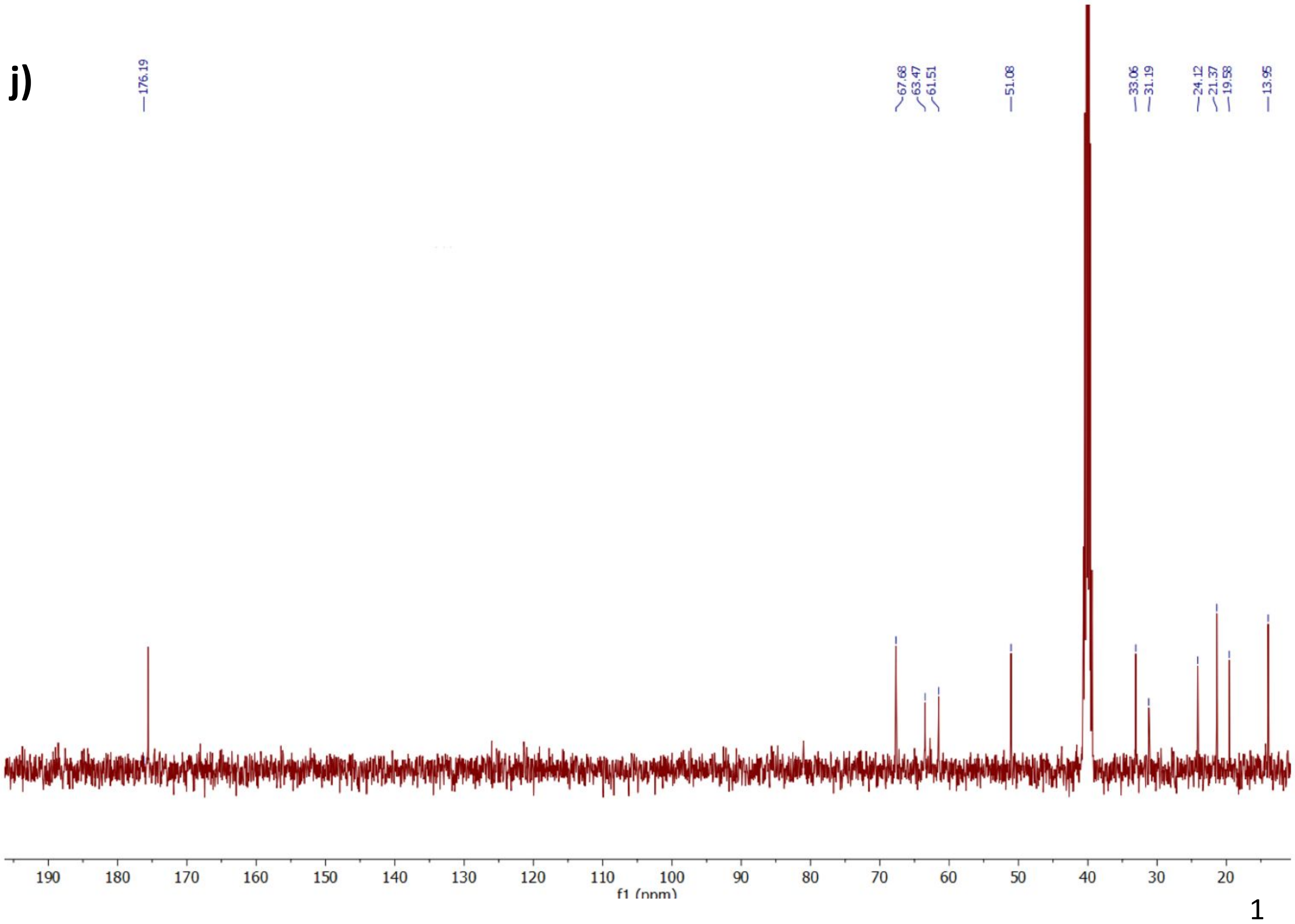
176.19
67.68
63.47
61.51
51.08
33.06
31.19
24.12
21.37
19.58
13.95
190 180 170 160 150 140 130 120 110 100 90 80 70 60 50 40 30 20
f1 (ppm)

k)

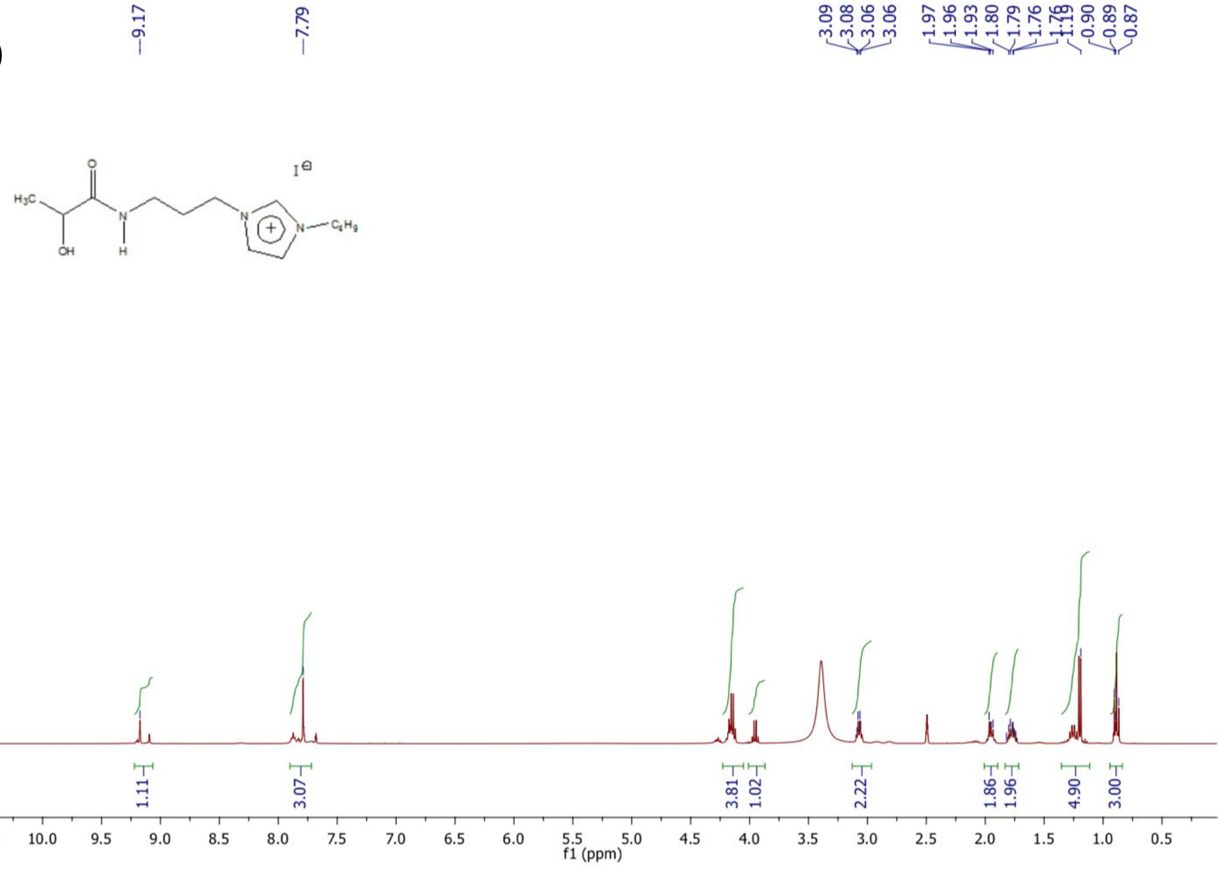
9.17
7.79
3.09 3.08 3.06 3.06
1.97 1.96 1.93 1.80 1.79 1.76 1.76 1.19 0.90 0.89 0.87
1.11 3.07 3.81 1.02 2.22 1.86 1.96 4.90 3.00
f1 (ppm)

l)

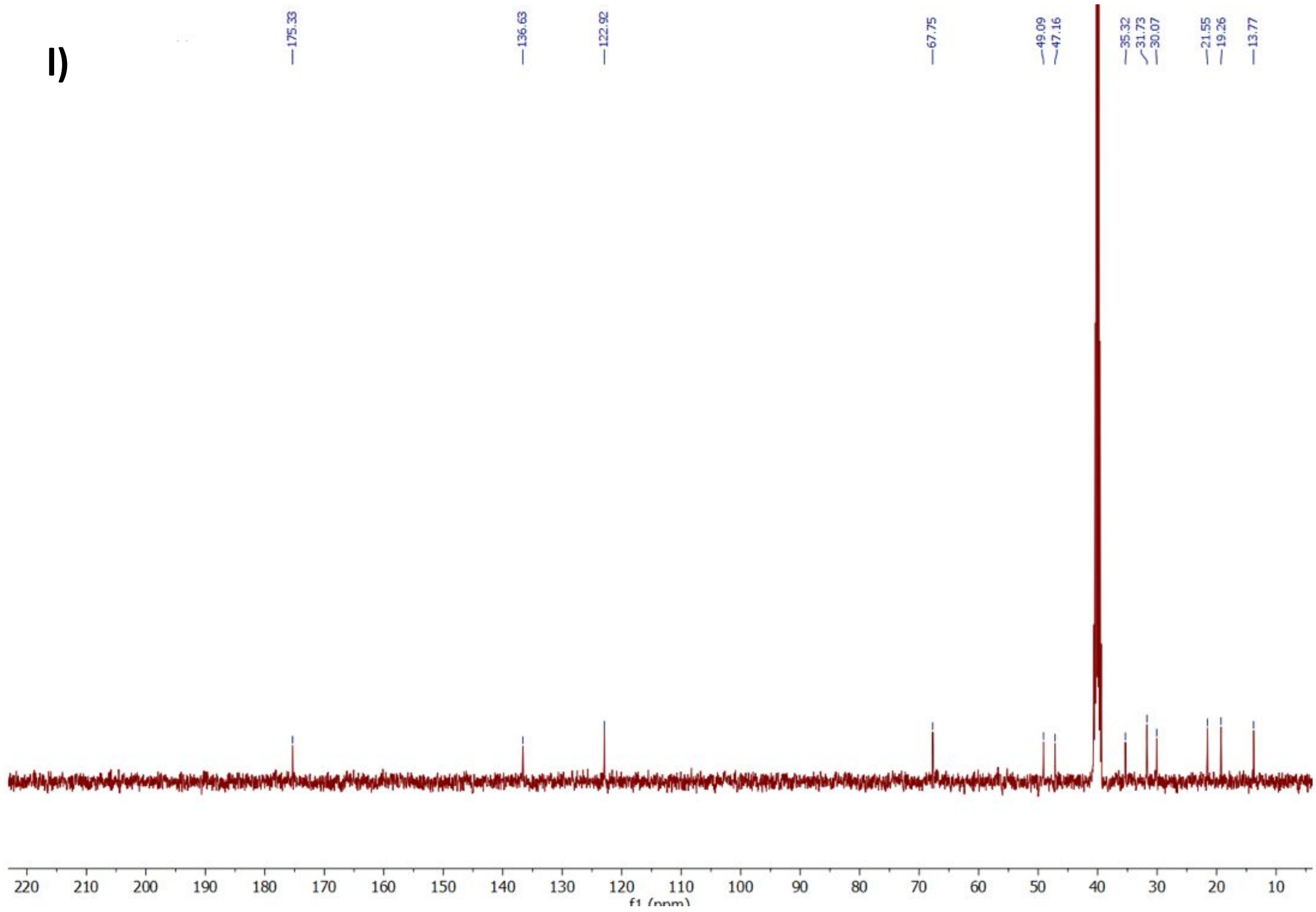
175.33
136.63
122.92
67.75
49.09 47.16
35.32 31.73 30.07
21.55 19.26
13.77
f1 (ppm)

m)

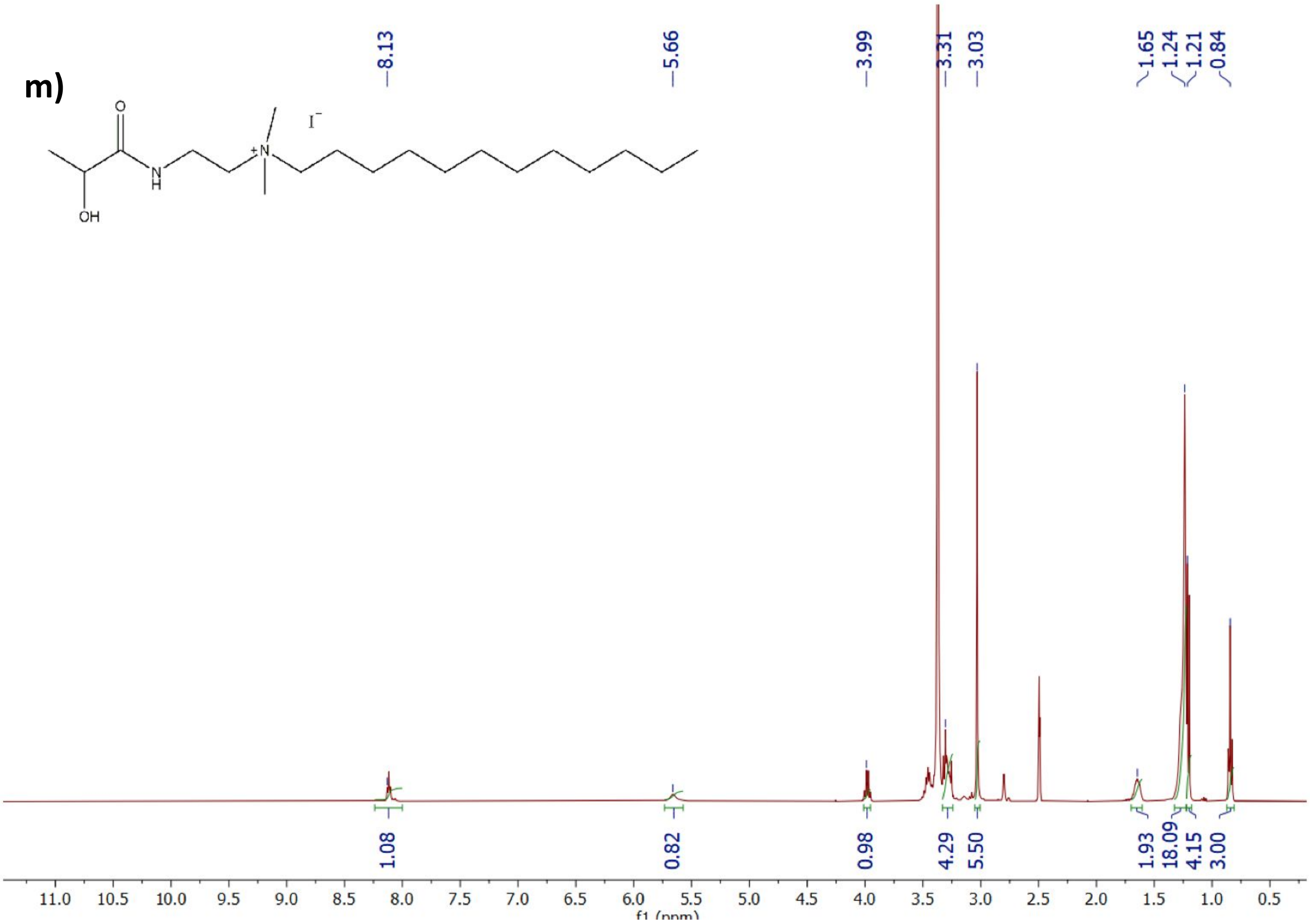

O
H
N
N
I
OH
8.13
5.66
3.99
3.31
3.03
1.65
1.24
1.21
0.84
1.08
0.82
0.98
4.29
5.50
1.93
18.09
4.15
3.00
11.0 10.5 10.0 9.5 9.0 8.5 8.0 7.5 7.0 6.5 6.0 5.5 5.0 4.5 4.0 3.5 3.0 2.5 2.0 1.5 1.0 0.5
f1 (ppm)

n)

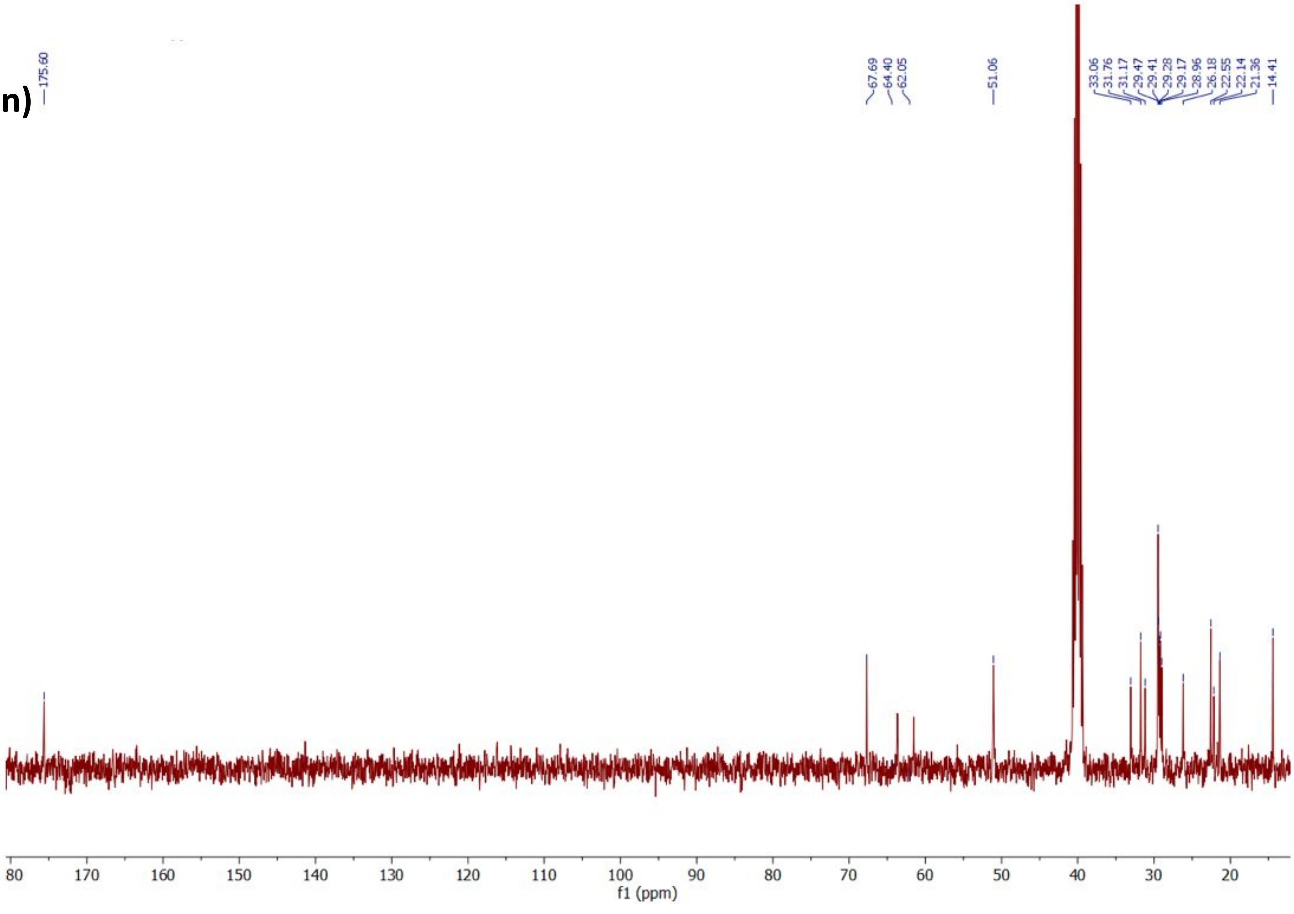

175.60
67.69
64.40
62.05
51.06
33.06
31.76
31.17
29.47
29.41
29.28
29.17
28.96
26.18
22.55
22.14
21.36
14.41
80 170 160 150 140 130 120 110 100 90 80 70 60 50 40 30 20
f1 (ppm)

o)

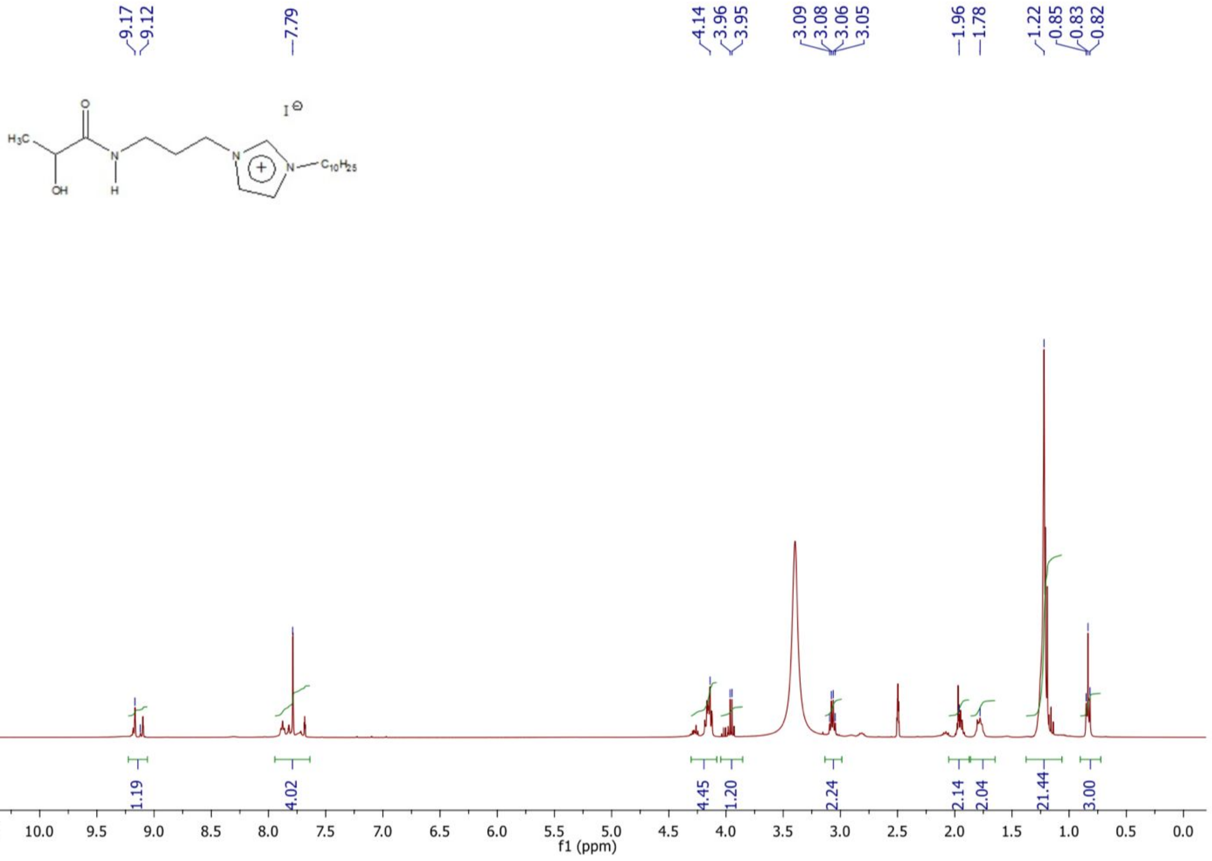
9.17
9.12
7.79
4.14
3.96
3.95
3.09
3.08
3.06
3.05
1.96
1.78
1.22
0.85
0.83
0.82
I⊖
H₃C
O
N
H
OH
N
+
N
C₁₀H₂₅
1.19
4.02
4.45
1.20
2.24
2.14
2.04
21.44
3.00
f1 (ppm)

p)

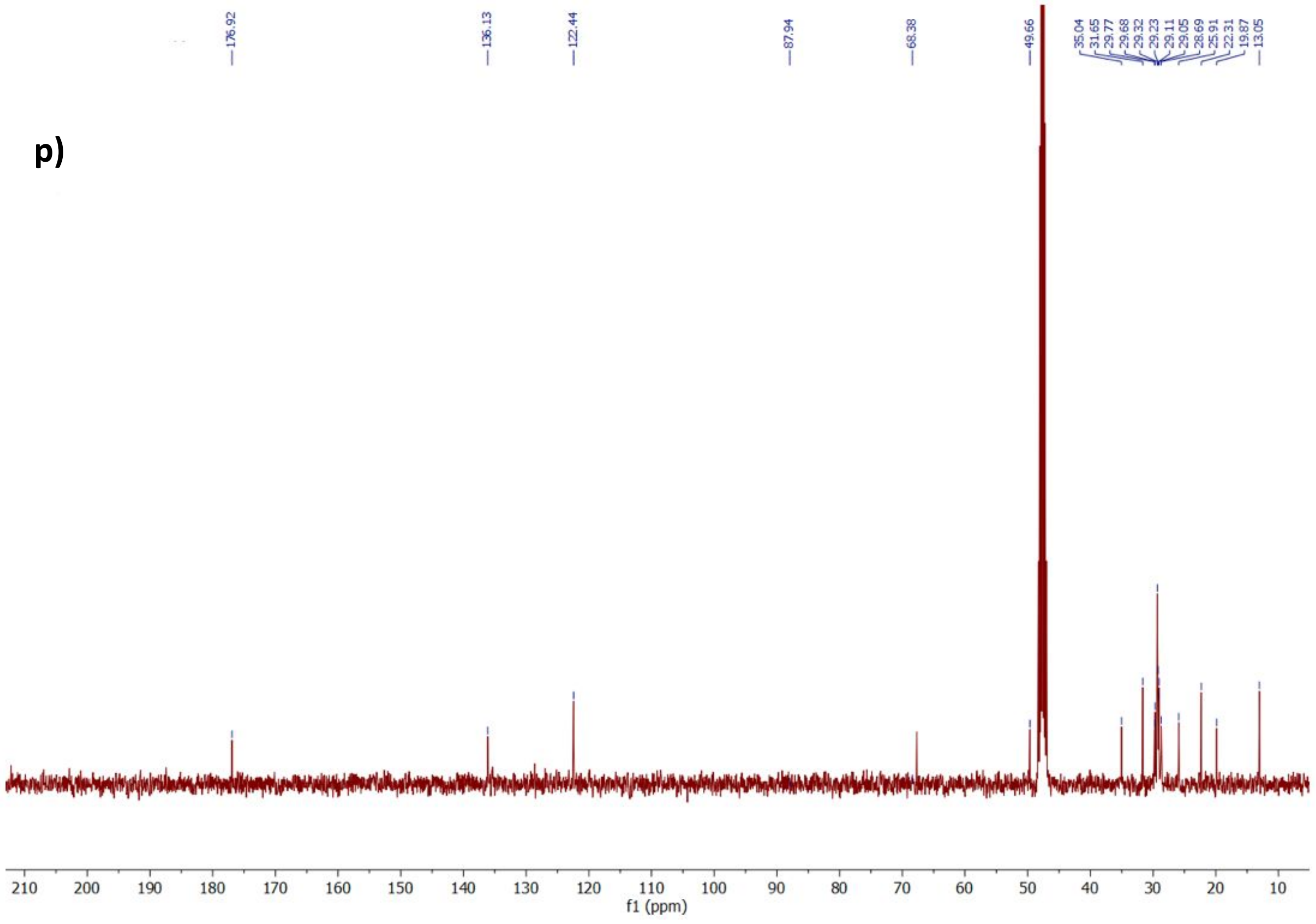
176.92
136.13
122.44
87.94
68.38
49.66
35.04
31.65
29.77
29.68
29.32
29.23
29.11
29.05
28.69
25.91
22.31
19.87
13.05
f1 (ppm)

q)

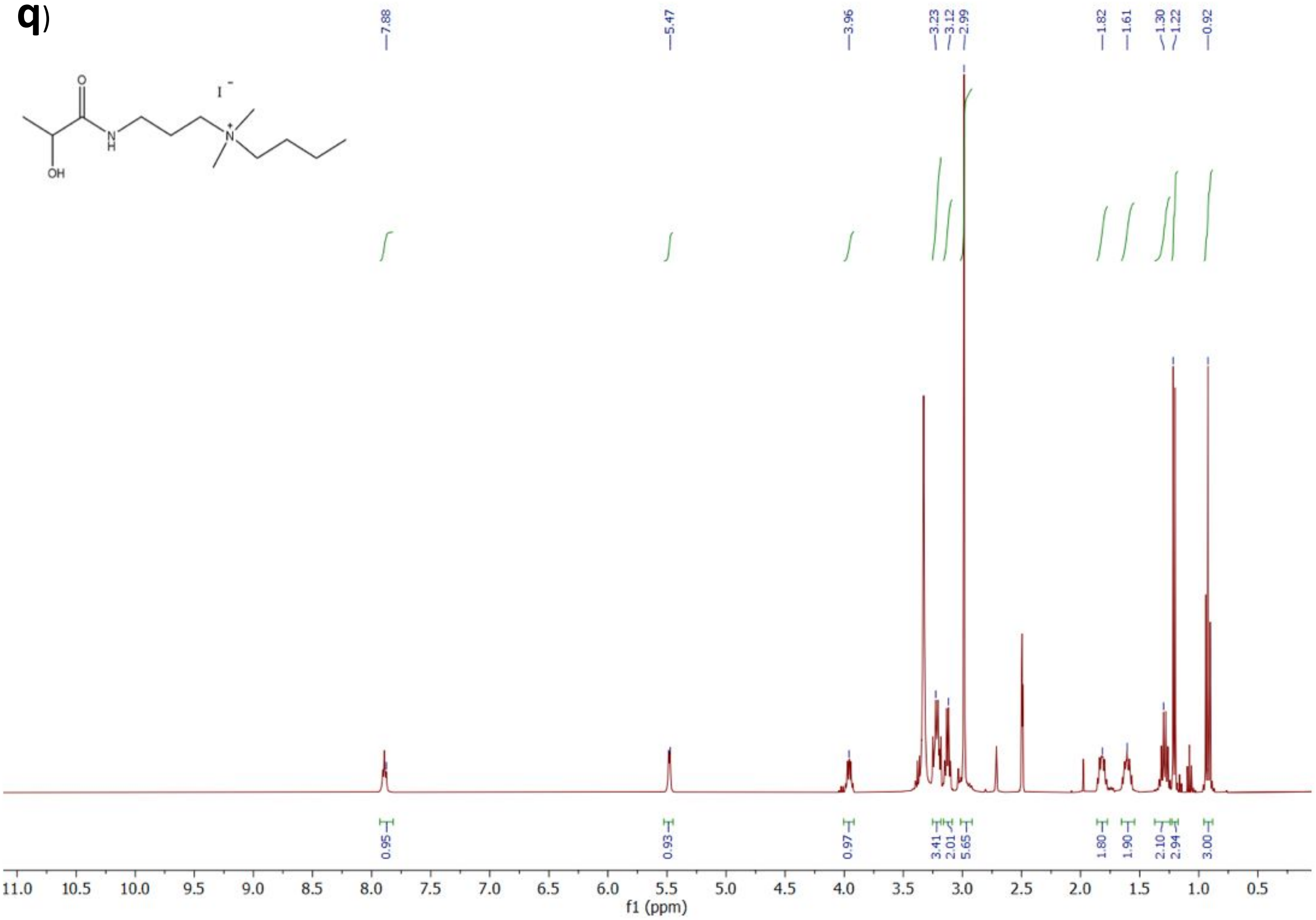

I⁻
OH
O
H
N
7.88
5.47
3.96
3.23
3.12
2.99
1.82
1.61
1.30
1.22
0.92
0.95
0.93
0.97
3.41
2.01
5.65
1.80
1.90
2.10
2.94
3.00
f1 (ppm)

r)

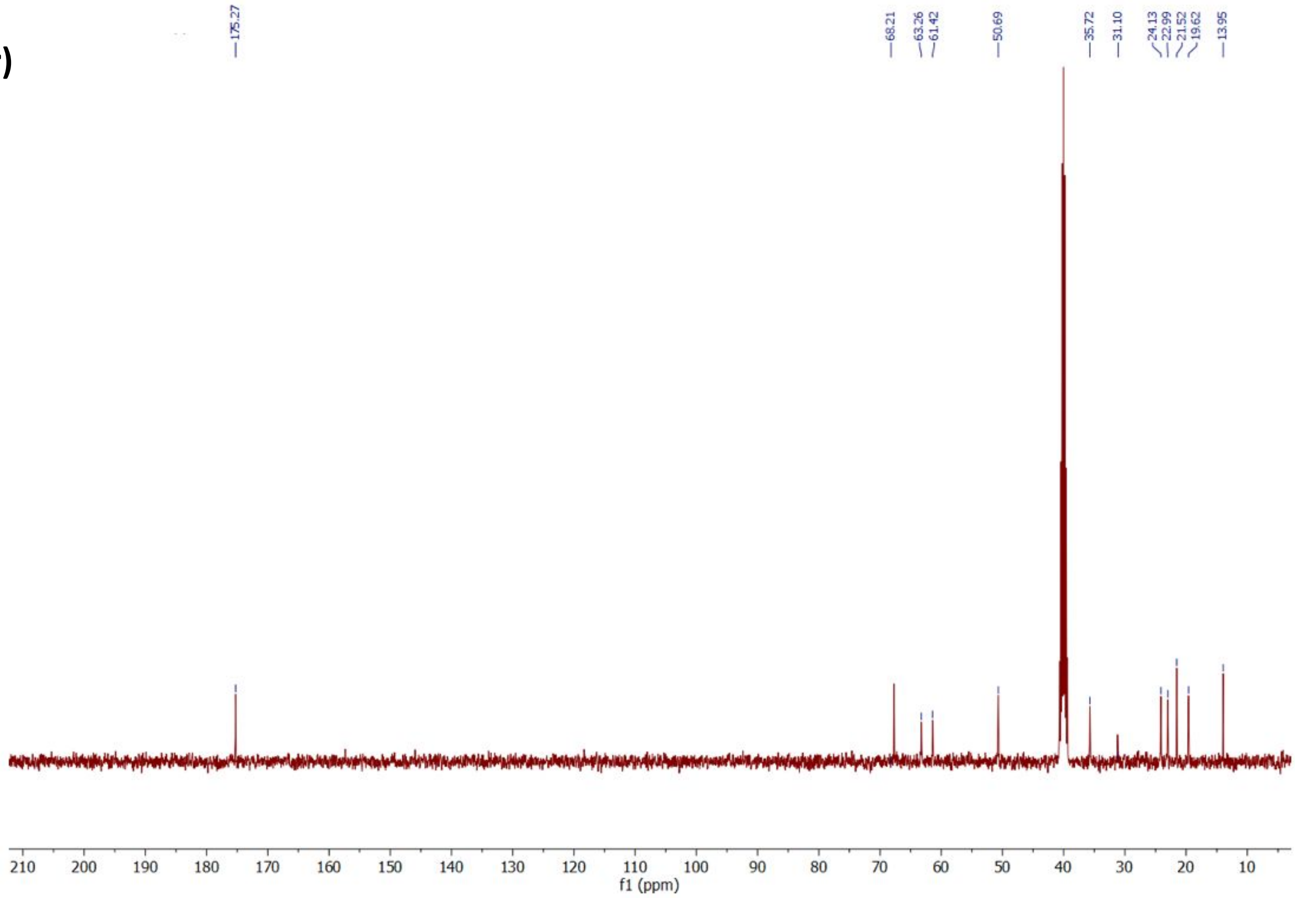

175.27
68.21
63.36
61.42
50.69
35.72
31.10
24.13
22.99
21.52
19.62
13.95
f1 (ppm)

s)

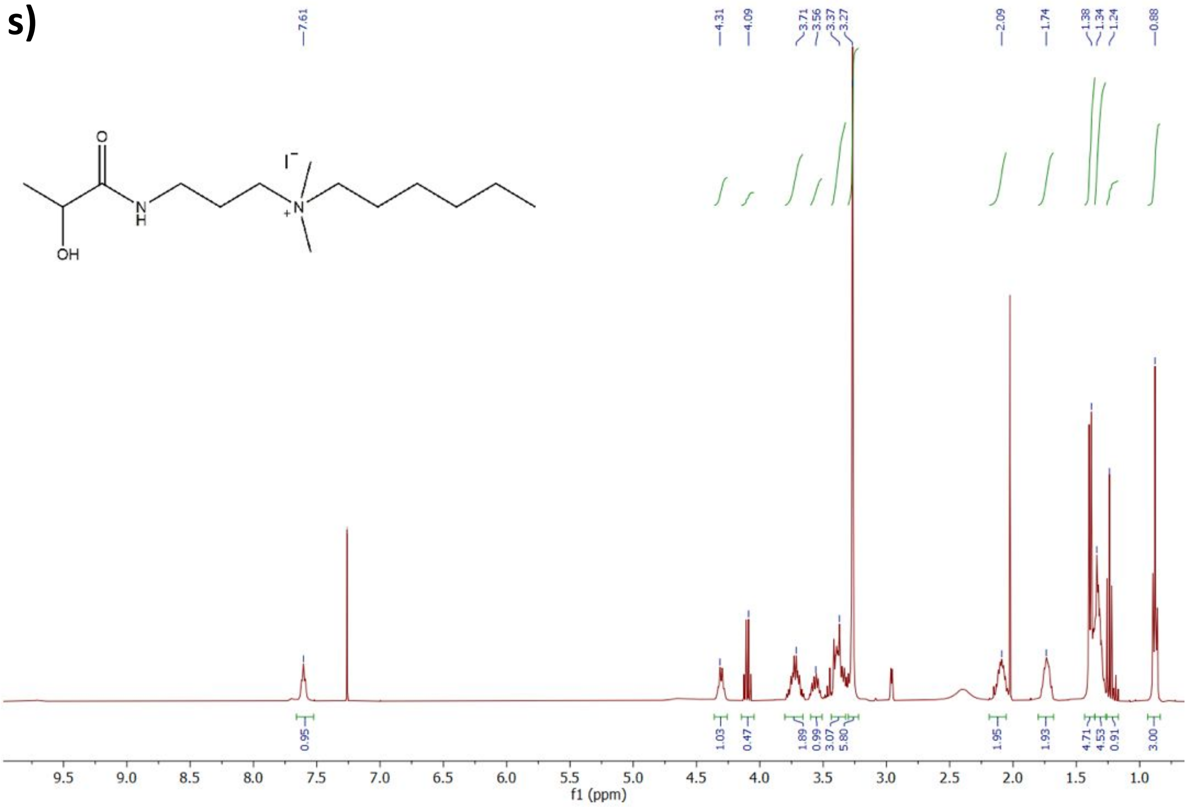
7.61
4.31
4.09
3.71
3.56
3.37
3.27
2.09
1.74
1.38
1.34
1.24
0.88
I⁻
O
N
H
N
+
OH
0.95
1.03
0.47
1.89
0.99
3.07
5.80
1.95
1.93
4.71
4.53
0.91
3.00
9.5 9.0 8.5 8.0 7.5 7.0 6.5 6.0 5.5 5.0 4.5 4.0 3.5 3.0 2.5 2.0 1.5 1.0
f1 (ppm)

t)

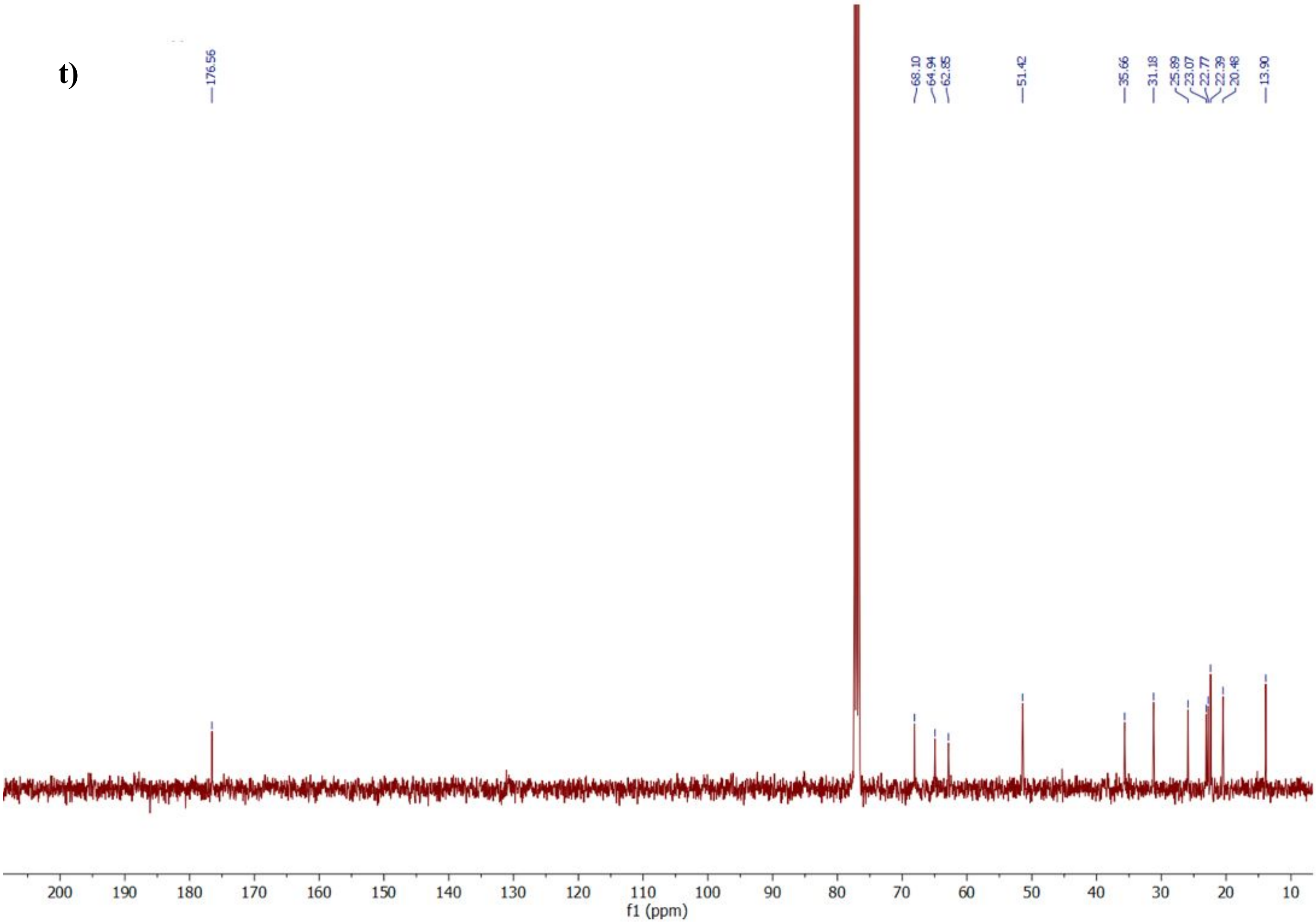
176.56
68.10
64.94
62.85
51.42
35.66
31.18
25.89
23.07
22.77
22.39
20.48
13.90
200 190 180 170 160 150 140 130 120 110 100 90 80 70 60 50 40 30 20 10
f1 (ppm)

u)

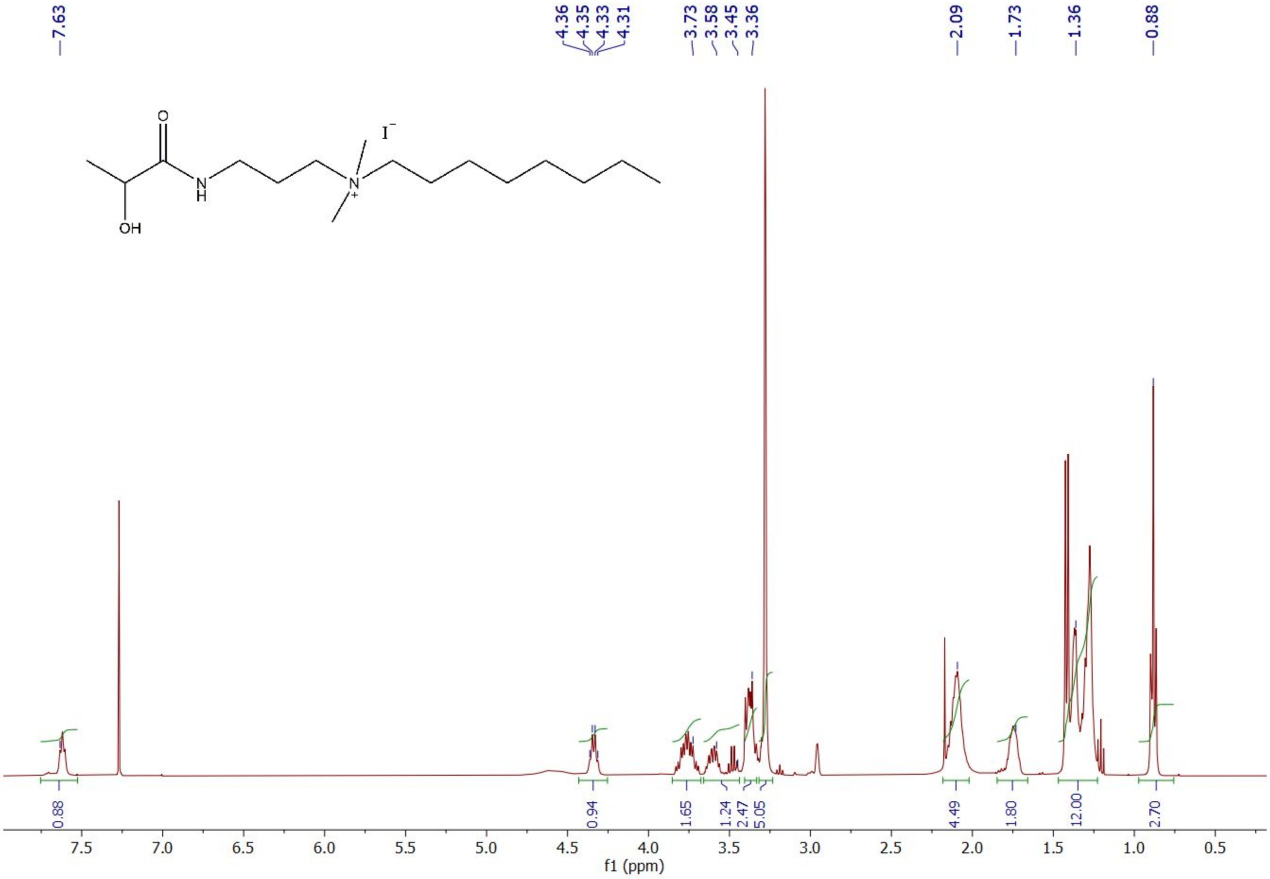
7.63
4.36
4.35
4.33
4.31
3.73
3.58
3.45
3.36
2.09
1.73
1.36
0.88
O
N
H
OH
N
+
I⁻
0.88
0.94
1.65
1.24
2.47
5.05
4.49
1.80
12.00
2.70
7.5 7.0 6.5 6.0 5.5 5.0 4.5 4.0 3.5 3.0 2.5 2.0 1.5 1.0 0.5
f1 (ppm)

v)

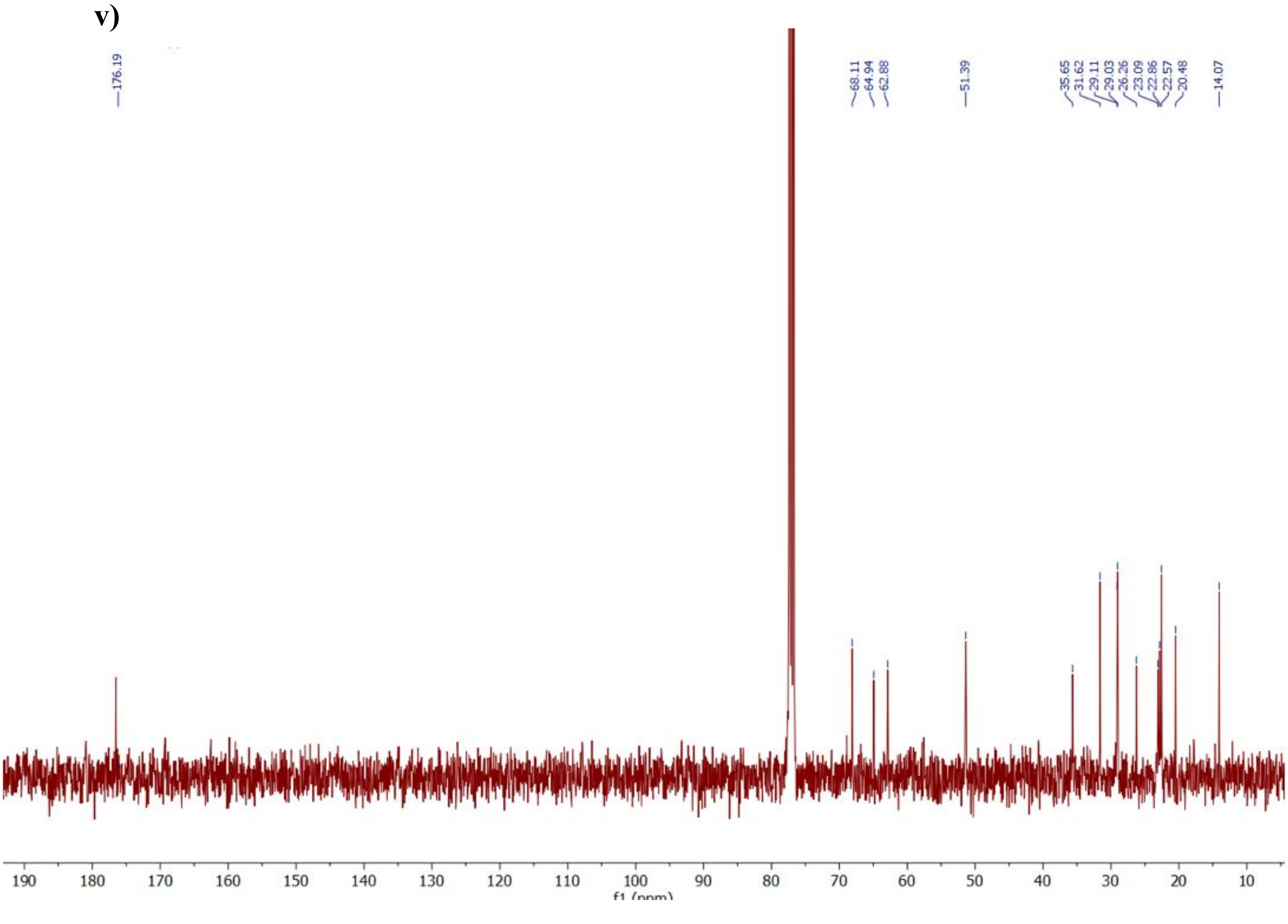
176.19
68.11
64.94
62.88
51.39
35.65
31.62
29.11
29.03
26.26
23.09
22.86
22.57
20.48
14.07
190 180 170 160 150 140 130 120 110 100 90 80 70 60 50 40 30 20 10

w)

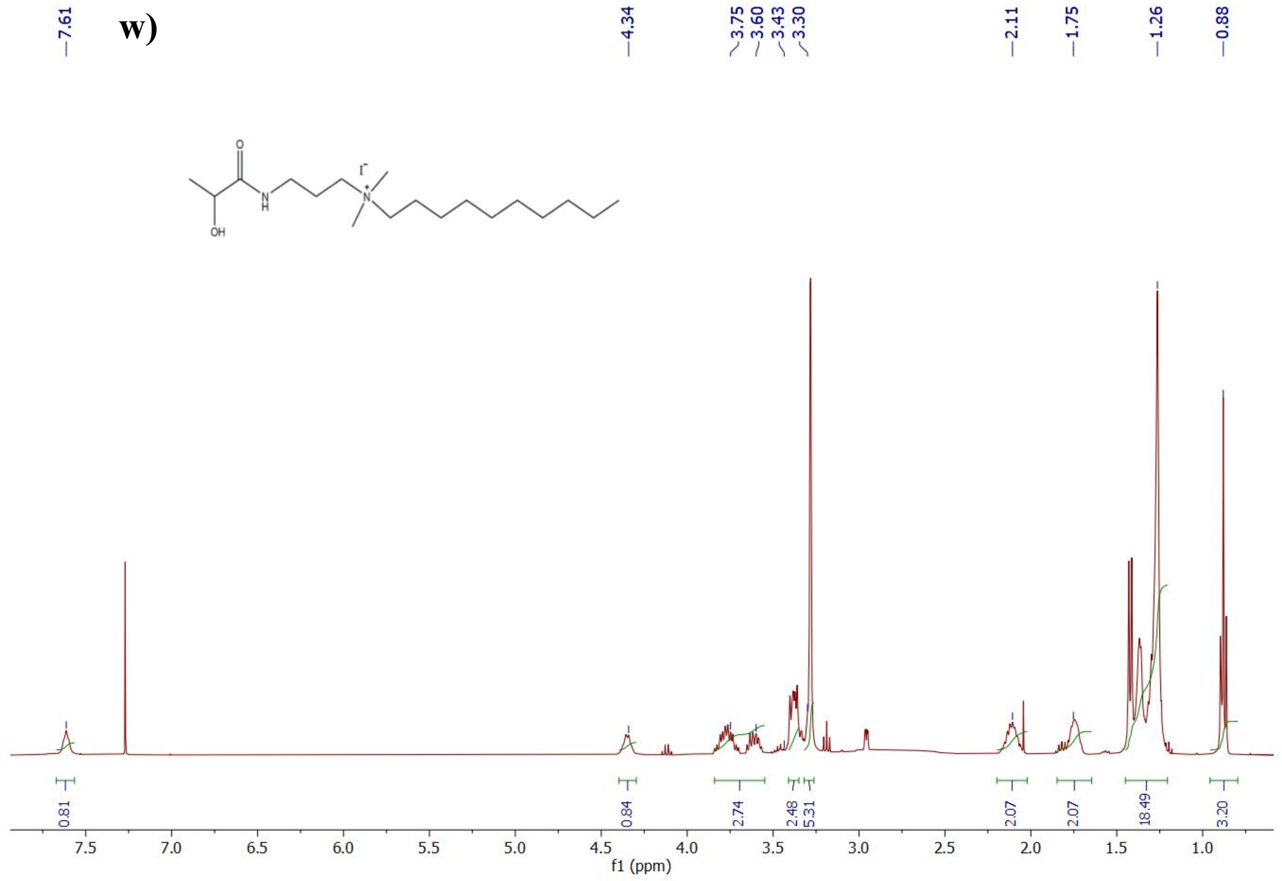
7.61
4.34
3.75
3.60
3.43
3.30
2.11
1.75
1.26
0.88
0.81
0.84
2.74
2.48
5.31
2.07
2.07
18.49
3.20
7.5 7.0 6.5 6.0 5.5 5.0 4.5 4.0 3.5 3.0 2.5 2.0 1.5 1.0
f1 (ppm)

x)

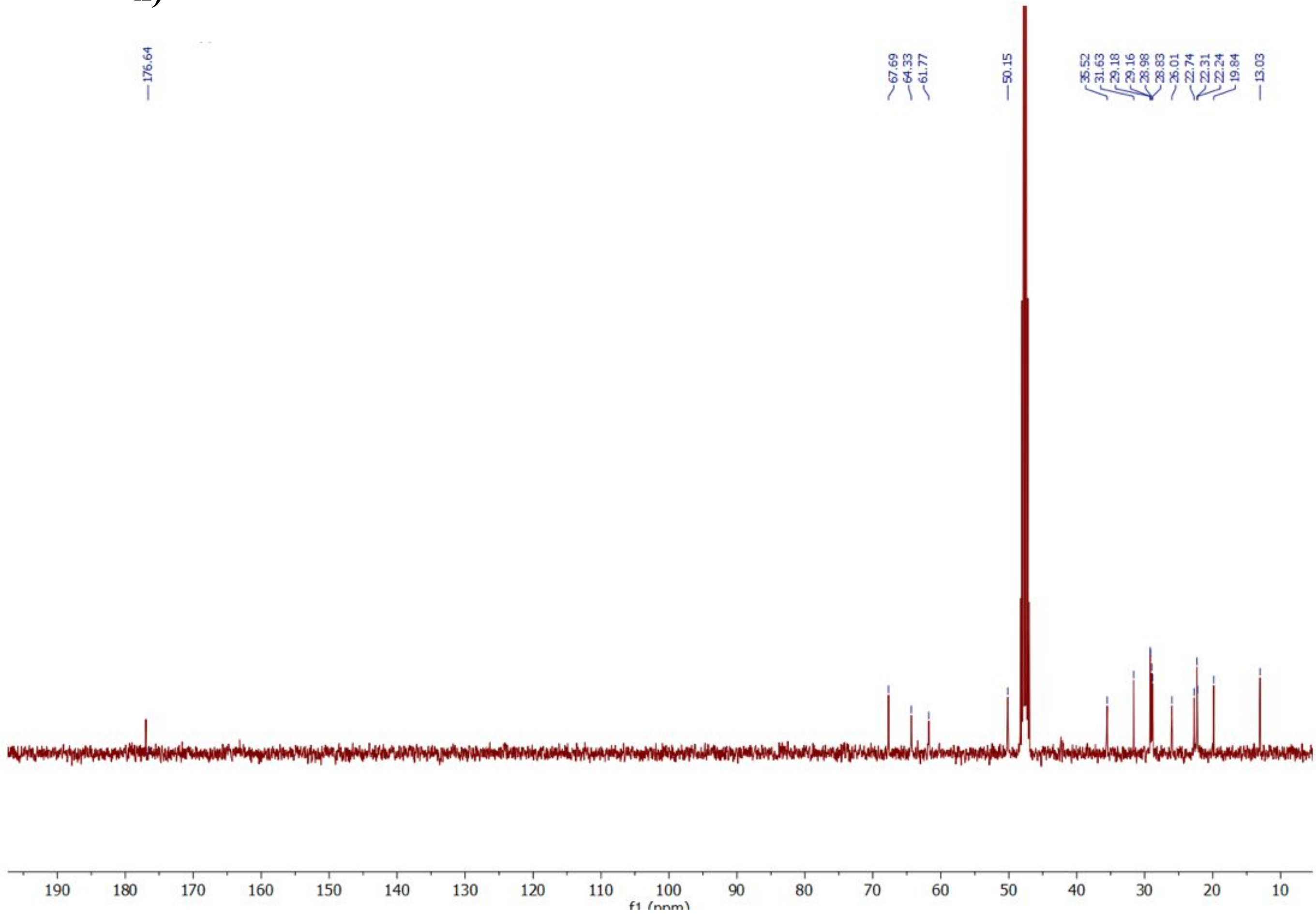
176.64
67.69
64.33
61.77
50.15
35.52
31.63
29.18
29.16
28.98
28.83
26.01
22.74
22.31
22.24
19.84
13.03
190 180 170 160 150 140 130 120 110 100 90 80 70 60 50 40 30 20 10

y)

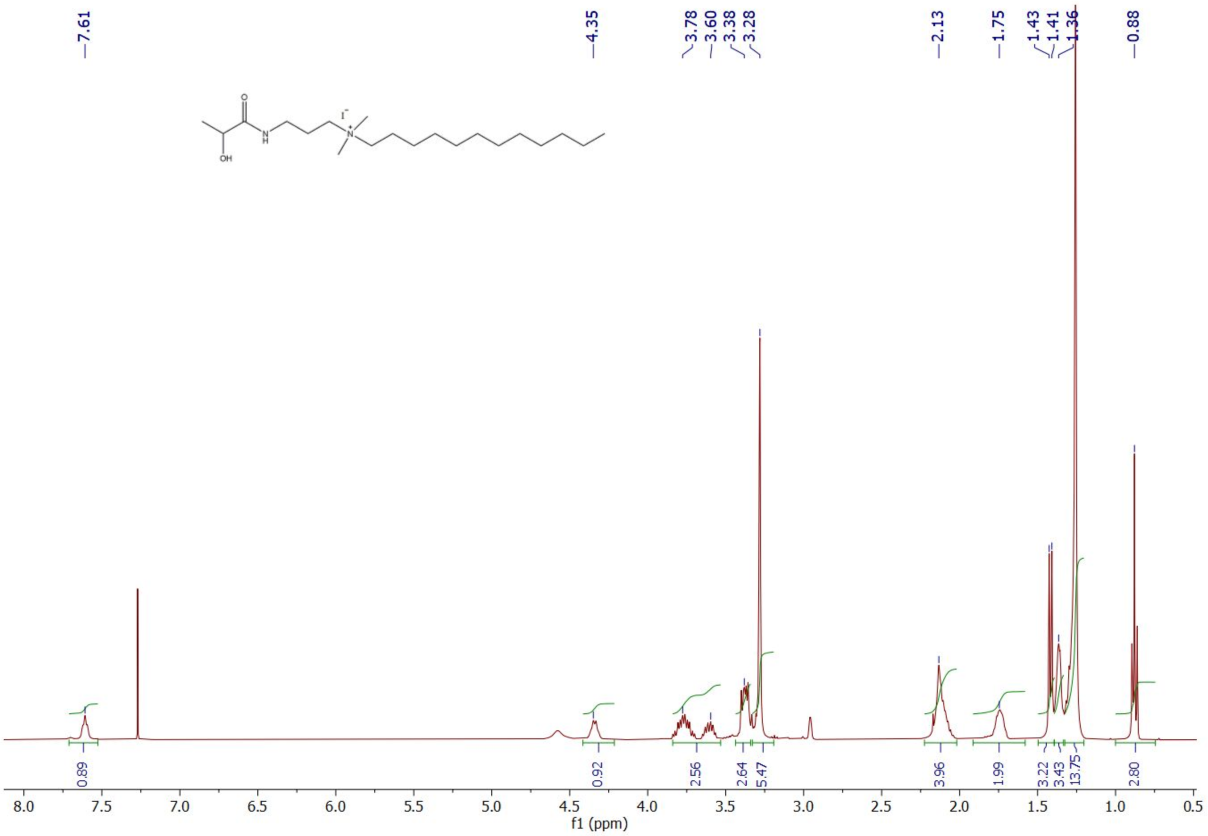

z)

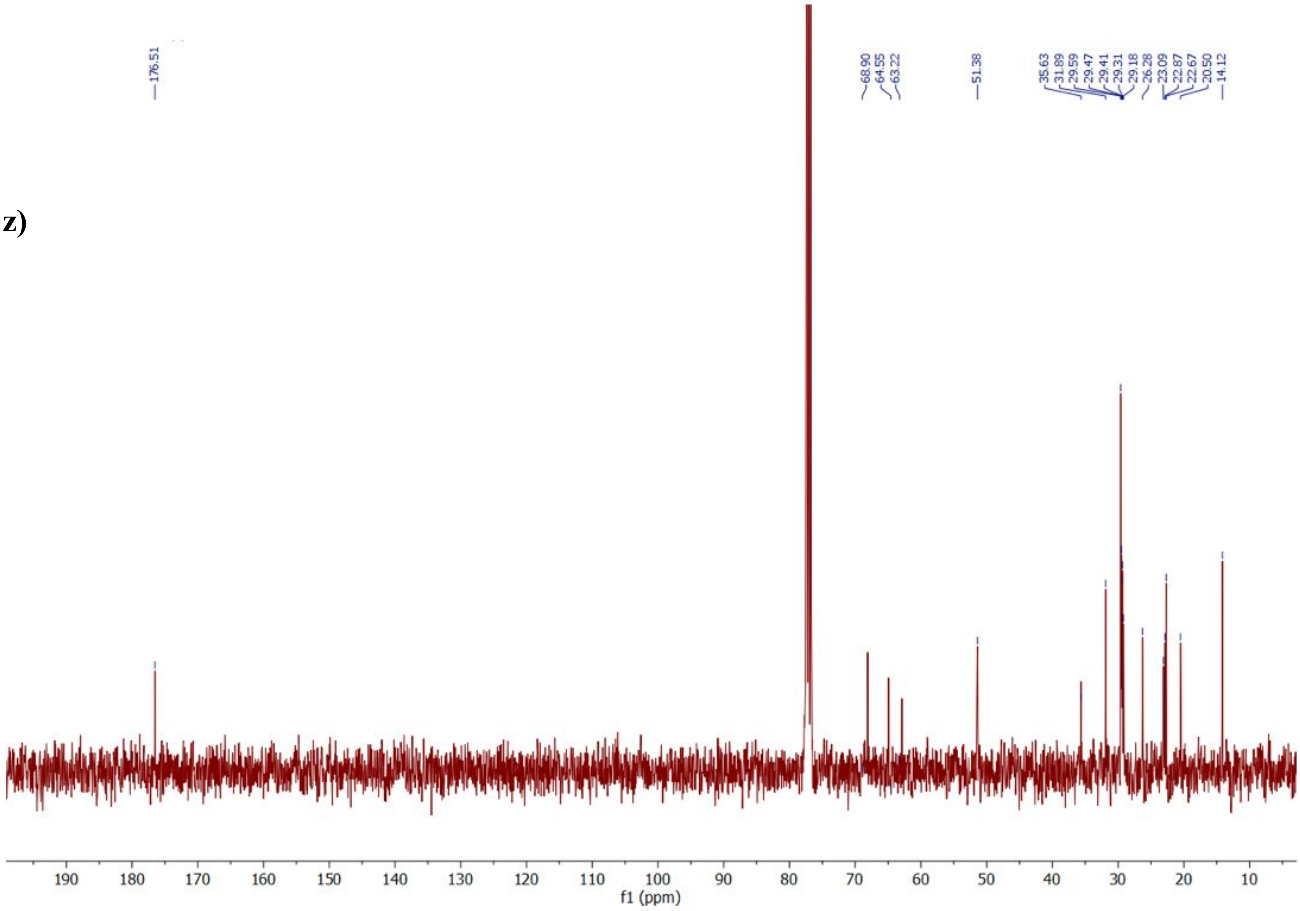

Figure S3. ^{1}H and ^{13}C NMR spectra of a)-d) lactamides, e) and f) **[N_{113}-Lac-4][I],** g) and h) **[N_{113}-Lac-12][Br],** i) and j) **[N_{112}-Lac-4][I],** k) and l) **[Im_3-Lac-4][I],** m) and n) **[N_{112}-Lac-12][I],** o) and p) **[Im_3-Lac-12][I],** q) and r) **[N_{113}-Lac-4][Br],** s) and t) **[N_{113}-Lac-6][I],** u) and v) **[N_{113}-Lac-8][I],** w) and x) **[N_{113}-Lac-10][I],** y) and z) **[N_{113}-Lac-12][I].**

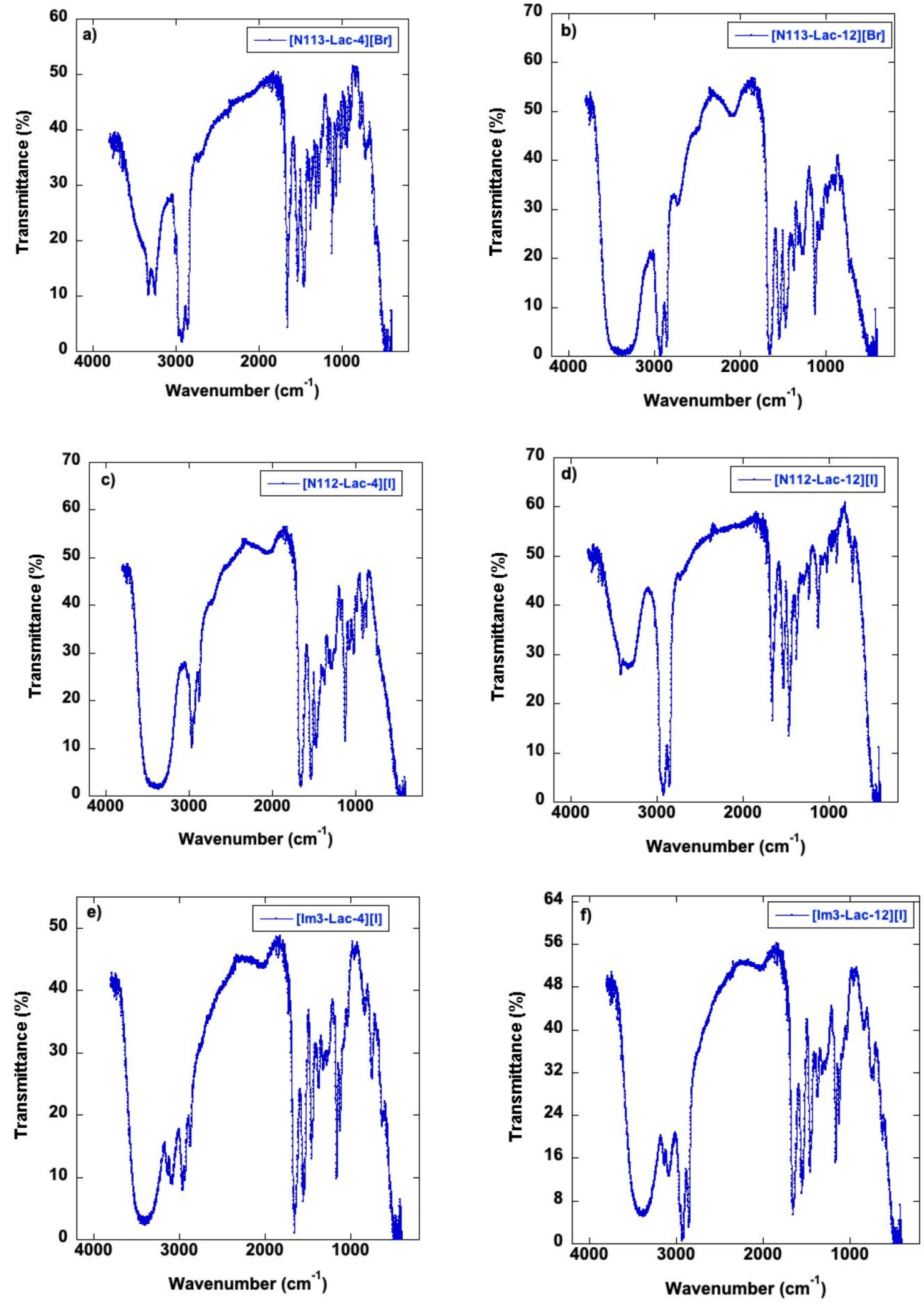
a)
[N113-Lac-4][Br]
b)
[N113-Lac-12][Br]
c)
[N112-Lac-4][I]
d)
[N112-Lac-12][I]
e)
[Im3-Lac-4][I]
f)
[Im3-Lac-12][I]
Transmittance (%)
Wavenumber (cm^{-1})

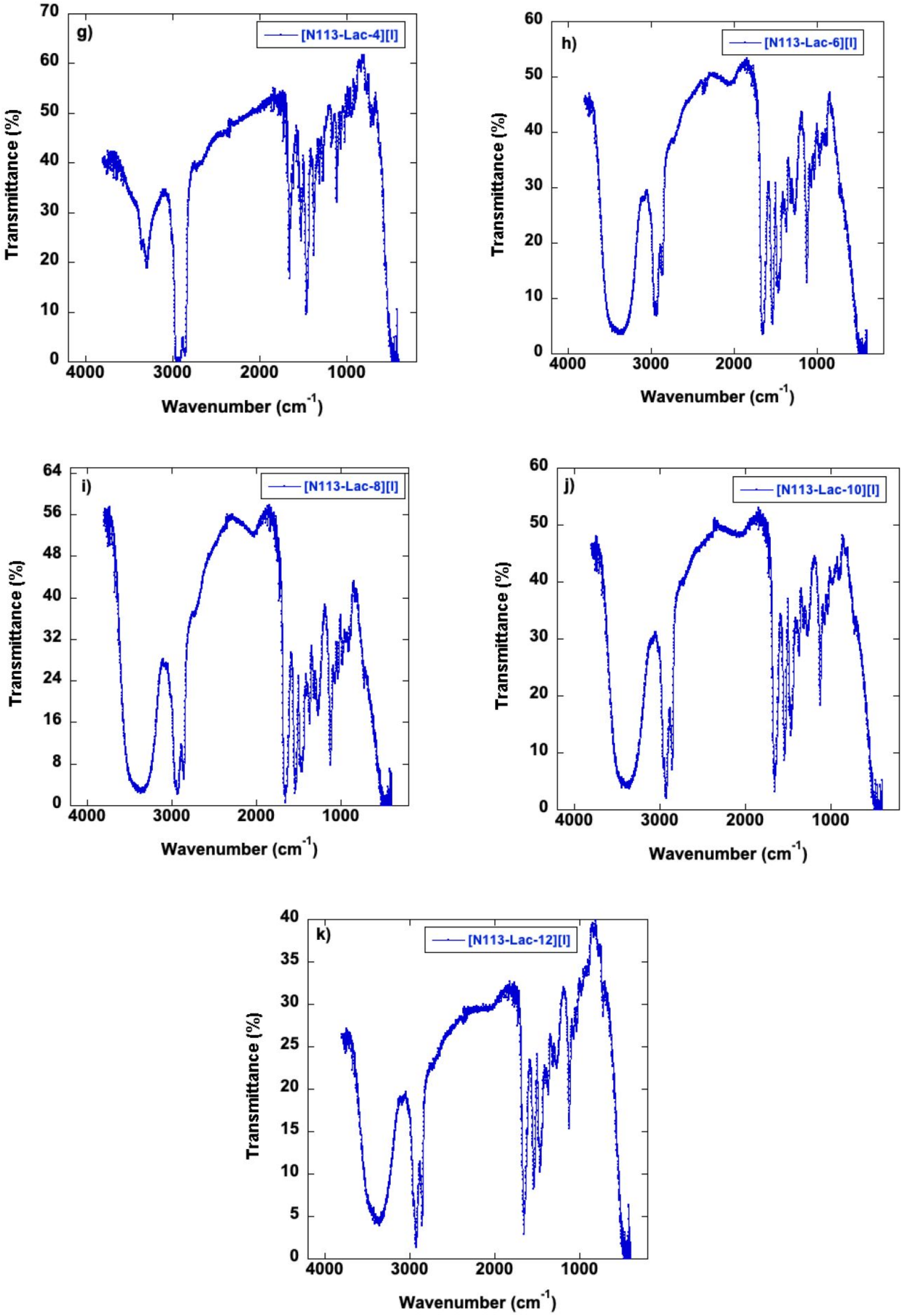

Figure S4. FTIR spectra of) a) **[N_{113}-Lac-4][Br],** b) **[N_{113}-Lac-12][Br],** c) **[N_{112}-Lac-4][I],** d) **[N_{112}-Lac-12][I],** e) **[Im_3-Lac-4][I],** f) **[Im_3-Lac-12][I],** g) **[N_{113}-Lac-4][I],** h) **[N_{113}-Lac-6][I],** i) **[N_{113}-Lac-8][I],** j) **[N_{113}-Lac-110][I]** and k) **[N_{113}-Lac-12][I].**

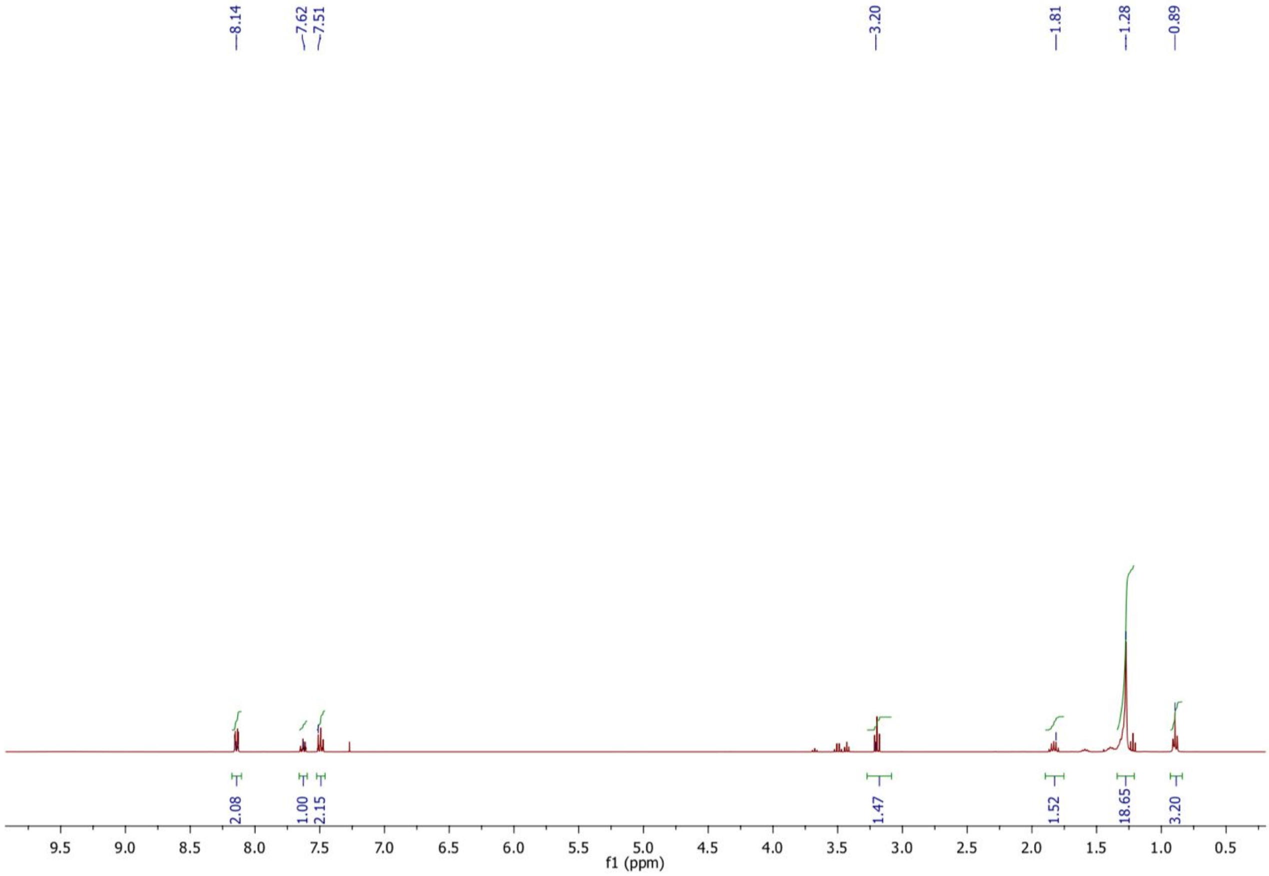

Figure S5. ^{1}H spectrum in $CDCl_3$ of the residue of alkylation, to recover iodododecane. Benzoic acid was added as internal standard.
